# ^17^O-EPR determination of the structure and dynamics of copper single-metal sites in zeolites

**DOI:** 10.1038/s41467-021-24935-7

**Published:** 2021-07-30

**Authors:** Paolo Cleto Bruzzese, Enrico Salvadori, Stefan Jäger, Martin Hartmann, Bartolomeo Civalleri, Andreas Pöppl, Mario Chiesa

**Affiliations:** 1 Felix Bloch Institute for Solid State Physics, Universität Leipzig, Leipzig, Germany; 2 Department of Chemistry and NIS Centre of Excellence, University of Turin, Torino, Italy; 3 Erlangen Center for Interface Research and Catalysis (ECRC), Erlangen, Germany

**Keywords:** Catalytic mechanisms, Heterogeneous catalysis, Porous materials

## Abstract

The bonding of copper ions to lattice oxygens dictates the activity and selectivity of copper exchanged zeolites. By ^17^O isotopic labelling of the zeolite framework, in conjunction with advanced EPR methodologies and DFT modelling, we determine the local structure of single site Cu^II^ species, we quantify the covalency of the metal-framework bond and we assess how this scenario is modified by the presence of solvating H_2_^16^O or H_2_^17^O molecules. This enables to follow the migration of Cu^II^ species as a function of hydration conditions, providing evidence for a reversible transfer pathway within the zeolite cage as a function of the water pressure. The results presented in this paper establish ^17^O EPR as a versatile tool for characterizing metal-oxide interactions in open-shell systems.

## Abstract (web summary)

The identification of catalytically active sites with atomic-scale precision occupies a central place in the theory and practice of heterogeneous catalysis. Here the authors assess the nature of the copper-oxygen bond in a Cu-CHA zeolite and recover the microscopic structure of single-metal sites.

## Introduction

Copper-exchanged zeolites have been in the focus of comprehensive studies for decades^1,2^ and are a subject of evergreen interest in catalysis due to applications ranging from NO_x_ removal^3,4^ to the direct conversion of methane to methanol^5–7^. Among other systems, the Cu-Chabazite (Cu-CHA) redox catalyst is particularly attractive as it couples industrial and environmental relevance—a Cu-CHA catalyst for diesel engine exhaust was commercialized since 2008—and structural simplicity. Cu-CHA catalysts have been widely investigated and it is agreed that isolated Cu^II^ ions at ion-exchange positions are the active sites for the NH_3_-SCR reaction^8^. However, the exact nature of the active sites and the impact of e.g. the Si/Al ratio and the copper content on the catalytic performance are still under debate. These make it an ideal model system to address fundamental questions of structure–performance relationships in the general context of metal-exchanged zeolite catalysis^1^.

The high activity of copper-containing zeolites in oxidation and reduction reactions is typically associated with the redox transformations of copper species. These range from isolated single ion sites^9^ to polynuclear species^10^, all featuring unsaturated coordination and the possibility for adsorption of small molecules. One crucial aspect to understand and control the catalytic potential of such species is the detailed knowledge of their structure and of the intimate features of chemical bonding, which include covalency, ionicity, electron and spin delocalization.

For paramagnetic Cu^II^ species (*S* = 1/2) the sensitivity and selectivity of EPR techniques are in principle ideally suited to obtain detailed information on the topological distribution and the nature of the chemical bonding of Cu^II^ species in zeolites and indeed EPR has been abundantly used to investigate such systems^11–20^. However, the most revealing and important piece of information that can be extracted from this technique, the detection of hyperfine couplings (a measure of the interaction between the electron spin and the nuclear spin of atoms in contact with the unpaired electron) for the coordinating oxygen atoms, is currently missing.

The only magnetic isotope of oxygen is ^17^O (*I* = 5/2) but its natural abundance (0.037%) is by far lower than the value necessary to detect a hyperfine structure. The exploitation of hyperfine techniques for the investigation of the metal-oxygen bond requires therefore the isotopic enrichment of oxide solid systems that involves both cost and effort, which however can be very rewarding. Indeed ^17^O solid state NMR has revealed invaluable to address important issues in this context, providing unique insights into crucial aspects related to the local structure of aluminosilicate zeolites^21,22^. However, this approach cannot be applied to investigate the Cu–O interaction due to the paramagnetic nature of Cu^II^ ions. ^17^O EPR has been used in different context to derive structural information on paramagnetic species^23–28^. Here we show that the ^17^O isotopic labelling of the framework provides a unique source of information about the local binding environment of the active site enabling the rationalization of structure-property relationships in Cu-loaded zeolites under, for instance, different hydration conditions. Water has been shown to play a strong effect on the reactivity of Cu species in zeolites promoting specific reaction pathways^29–31^, promoting dynamic catalytic mechanisms at the cross road between homogeneous and heterogeneous catalysis^32–34^.

The ^17^O resonance from oxygen atoms directly bound to Cu^II^ species in a low copper loaded Chabazite is reported. This enables the identification of two specific binding sites which are selectively populated as a function of the hydration conditions. By the selective isotopic labelling of the zeolite framework with ^17^O and employing advanced EPR methodologies in conjunction with DFT modelling, we have been able to obtain exquisite details on the nature of the Cu interaction with the oxygen donor atoms of the zeolite framework and of solvating water molecules. We demonstrate that the measured ^17^O hyperfine couplings provide a so far unexplored and effective handle to obtain a detailed understanding of the Cu–O bond, to assess the siting of Al in the most stable Cu coordination and to follow the migration of Cu^II^ species across the zeolite channels as a function of hydrating conditions.

## Results and discussion

### Structure and dynamics of isolated Cu^II^ ions

**Fig. 1:**
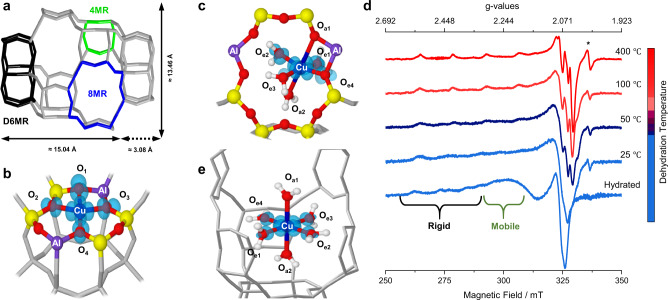
EPR spectra and structural models of Cu^II^ single sites in CHA. **a** Graphical representation of CHA framework. The fundamental units are highlighted with different colours. Spin density plots of dehydrated Cu^II^ ion sitting on 6MR and hydrated Cu^II^ complex attached to the framework in 8MR site are illustrated in (**b**, **c**), respectively. **d** X-band CW-EPR spectra recorded at room temperature of fully hydrated with H_2_^16^O Cu-CHA and dehydrated at increasing temperatures according to the procedure described in ‘Methods’ section. The hyperfine components of rigid and mobile species are indicated. The asterisk indicates a carbon radical signal. **e** Spin density plots of [Cu(H_2_O)_6_]^2+^ complex encapsulated in the largest Chabazite’s cage. The labels O_e*n*_ and O_a*n*_ refer to *n* equatorial and axial oxygen ligands, respectively.

The first key question we address is related to the structure and dynamics of Cu^II^ species as a function of hydration and their interaction with the zeolite framework. The fundamental building block of the CHA framework is a double six-membered ring (D6MR) unit disposed in layers according to an ABC stacking and linked by four-membered rings (4MRs). The interconnection of the D6MRs along the three dimensions generates eight-membered rings (8MRs) units (Fig. 1a). According to early reports^35–37^ and more recent studies^3,31^, the potential extra-framework sites of Cu cations are located on the window of D6MRs (Fig. 1b) or on 8MRs (Fig. 1c) with either two framework aluminium atoms (2Al) or one aluminium site (1Al) plus an extra-lattice OH^−^ ligand for charge compensation (for this latter case see Supplementary Note 3).

Continuous wave (CW)-EPR spectra of the Cu-exchanged CHA sample were recorded as a function of the dehydration temperature and show characteristic spectral patterns depending on the degree of hydration (Fig. 1d). The spectrum of the fully hydrated system is due to two *S* = 1/2 EPR signals, characteristic of Cu^II^ ion with a different local environment^3,11,38,39^. The room temperature (RT) spectrum shows the contribution of a motionally averaged and a rigid-limit anisotropic components, corresponding to a mobile solvated structure (Fig. 1e) and framework-bound Cu^II^ species (Fig. 1c), respectively (see Table 1 and for further details on low temperature CW-experiments, see Supplementary Fig. 1).

**Table 1:** Experimental Cu **g**- and **A**-tensors obtained from the simulations of the CW-EPR spectra recorded at 77 K.

Samples	Weight	*g*_*⊥*_	*g*_*//*_	*A*_*⊥*_	*A*_*//*_
Hydrated	55% A	2.070 ± 0.005	2.415 ± 0.001	30 ± 10	400 ± 5
	45% B	2.065 ± 0.006	2.370 ± 0.002	30 ± 10	450 ± 4
Dehydrated 400 °C	85% C	2.058 ± 0.002	2.355 ± 0.001	30 ± 10	462 ± 5
	15% D	2.062 ± 0.003	2.320 ± 0.001	30 ± 10	487 ± 4

Only the absolute values of the hyperfine components are extracted from the spectra. Hyperfine couplings are given in units of MHz.

The progressive dehydration of the sample (Fig. 1d) is accompanied by a narrowing of the spectral linewidth and disappearance of the motionally averaged component in the RT CW-EPR spectrum. A sharp signal at *g* = 2.0028 (asterisk in Fig. 1d) is observed in the dehydrated sample, which is often present even in the most careful calcination protocols and assigned to carbon radicals deriving from carbonaceous residues in the zeolite framework^40^. After dehydration at 400 °C the overall EPR spectral intensity is reduced of about 40% with respect to the fully hydrated system (see Supplementary Fig. 1). The loss of EPR intensity during the sample activation is a well-known phenomenon, which strongly depends on the Si/Al and Cu/Al ratios^41,42^. Dramatic losses of over 80% have been reported for Cu-CHA with Si/Al = 14 and Cu/Al in the range 0.1–0.4^39,42,43^. The limited reduction we have observed is consistent with the low Cu/Al = 0.005 ratio and is most likely associated to the presence of residual carbonaceous impurities (*g* = 2.0028 carbon radical signal, Fig. 1d), which act as reducing agents^44,45^. Although a contribution from the so-called ‘autoreduction’ mechanism^42,46^ cannot be excluded, this is expected to be relevant at higher Cu/Al ratios as it relies on the condensation of neighbouring hydroxyl bridged [CuOH]^+^ species whose presence is limited by the low Cu loading considered in this work^31^.

To summarize, the analysis of the CW-EPR spectra as a function of the sample dehydration evidences the presence of at least two Cu^II^ species characterized by distinctively different spin-Hamiltonian parameters, which change as a function of the dehydration treatment in line with previous reports^47^. In the case of the hydrated sample, two species with nearly equal abundance are present (Table 1). One such species (A in Table 1) shows spin-Hamiltonian parameters typical for [Cu(H_2_O)_6_]^2+^ complexes (Fig. 1e)^48,49^. The other species (B in Table 1) has spin-Hamiltonian parameters consistent with a tetragonally elongated 6-coordination, i.e. with a distorted octahedral geometry (Fig. 1c)^11,50^. On the other hand, the spectrum of the fully dehydrated sample is dominated by a Cu^II^ species accounting for about 80% of the total EPR intensity with spin-Hamiltonian parameters (C in Table 1) agreeing with a tetragonal planar coordination of Cu^II^ (Fig. 1b)^18,38,51,52^. These configurations are supported by density functional theory (DFT) calculations (for further details on the computed EPR parameters, see Supplementary Tables 3, 6, and 7) that give the singly occupied molecular orbital (SOMO) as dominated by the Cu d_x_^2^_–y_^2^ orbital with participation of the 2p oxygen orbitals (Fig. 1c–e). The degree of covalency of the Cu–O bond (i.e. the oxygen contribution to the SOMO) will be addressed in the following, along with the detailed topological description of the Cu^II^ docking sites under specific hydration conditions.

### Geometrical and electronic structure through spin density studies

Most of the information relative to the topological distribution of the Cu^II^ species is hidden in the inhomogeneously broadened line of the CW-EPR spectrum and is related to the hyperfine interactions (hfi) with nearby magnetic nuclei (^1^H, ^27^Al and ^17^O). In order to obtain such fundamental knowledge for the structural characterization of Cu^II^ siting, hyperfine techniques (HYSCORE and ENDOR) were employed at X- and Q-band frequencies.

**Fig. 2:**
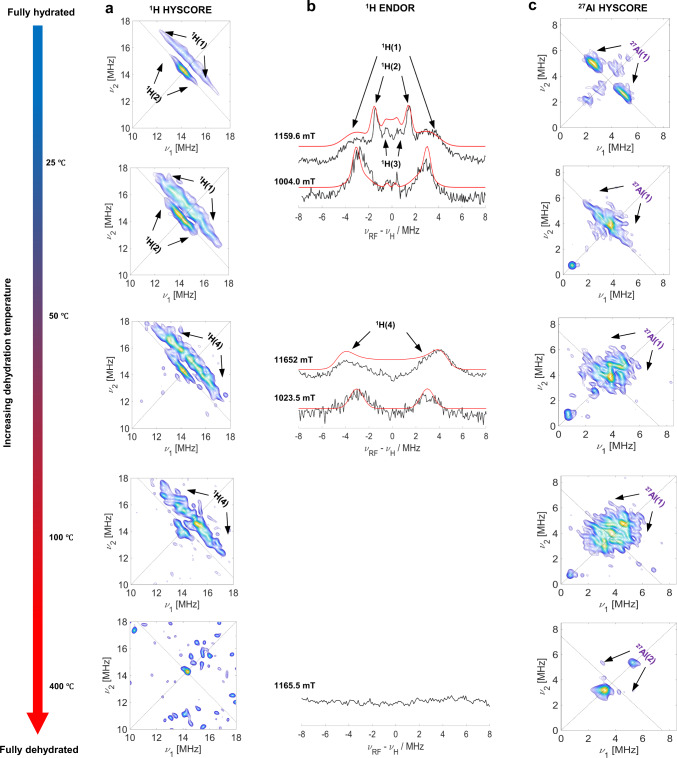
HYSCORE and ENDOR spectra of Cu-CHA at different dehydration stages. **a** X-band ^1^H HYSCORE spectra recorded at 4.5 K and obtained at the echo maximum intensity position of Cu-CHA gradually dehydrated. **b** Corresponding Q-band ^1^H Davies ENDOR spectra (black line) acquired at 20 K at the field indicated above the plot and their simulations (red line). **c** X-band ^27^Al HYSCORE spectra recorded at same experimental conditions of ^1^H HYSCORE spectra. ^1^H and ^27^Al signals are indicated by arrows. The parameters used in the simulation satisfy both HYSCORE and Davies ENDOR spectra.

The progressive dehydration of the sample was carefully followed by X-band HYSCORE and Q-band Davies ENDOR experiments. The results are shown in Fig. 2a–c where ^1^H HYSCORE, ^1^H ENDOR and ^27^Al HYSCORE experiments are shown for the same dehydration conditions reported in Fig. 1d.

The X-band ^1^H HYSCORE spectrum, correlates nuclear frequencies in the *α* and *β* electron spin manifolds that result in—for a set of magnetically equivalent protons featuring weak hyperfine couplings (2|*ν*_*H*_| > |*A*|)—ridges centered at the proton Larmor frequencies (*ν*_*H*_) and extension corresponding to the orientation-dependent hyperfine coupling constant (*A*). The same information can be retrieved by ENDOR experiments, which, under the same conditions, are characterized by a pattern consisting of a single pair of lines separated in frequency by *A* and mirrored about *ν*_*H*_. The ^1^H HYSCORE spectrum of the hydrated sample is characterized by two distinct ridges with maximum extension of the order of 9 and 3 MHz (Fig. 2a, at the top). These couplings are also confirmed by Q-band Davies ENDOR experiments (Fig. 2b, at the top). The field dependent experimental spectra are simulated exceedingly well considering two interacting protons with hyperfine coupling tensors (in MHz) of *A*_H(1)_ = [−5.5 −7.5 +8.5] and *A*_H(2)_ = [−2.7 −3.7 +7.3], whereby the maximum coupling of H(1) lies in the plane of the d_x_^2^_−y_^2^ orbital, while for H(2) is approximately perpendicular to it. The smaller hyperfine coupling also correlates with the larger axial Cu–O distance (around 0.23 nm with respect 0.19 nm of the equatorial Cu–O distance; distances obtained from periodic DFT geometry optimizations are listed in Supplementary Tables 1 and 5) of the axially coordinating water molecules. These values are typical for hexaaquacopper complexes (Fig. 1e) and were attributed by Pöppl and Kevan to equatorial and axial water molecules of the [Cu(H_2_O)_6_]^2+^ complex^49^. A very weakly coupled proton signal is also detected and labelled H(3) in Fig. 2, which we assign to second shell coordinating water molecules.

The intensity of the ^1^H HYSCORE ridges decreases until the signal completely disappears in the fully dehydrated state, probing the progressive dehydration of the sample. Correspondingly, the ^1^H ENDOR spectra show that the weakly coupled protons (H(2) and H(3)) are the first one to be lost at this dehydration stage. These couplings are amenable to axially coordinated water molecules (H(2)) or second shell coordinating H_2_O (H(3)), both displaying a weaker binding energy and therefore removed in the first stages of the dehydration process. On the other hand, H(4) nuclei possess hyperfine couplings and Euler angles similar to H(1), suggesting the stronger persistence of equatorial protons with respect to axial ones. When complete dehydration was achieved, the proton signals are no longer observed (Fig. 2a, b, at the bottom) proving that all coordinated water molecules were removed.

^27^Al HYSCORE spectra (Fig. 2c) display a pair of cross peaks centered at the Al Larmor frequency (*ν*_*Al*_) indicating a hyperfine interaction of the order of 3 MHz. The narrow shape of the cross peaks and absence of multiple quantum transitions indicate a low value of the nuclear quadrupole interaction (estimated to be of the order of *e*^*2*^*qQ/h* ≤ 4 MHz, see Supplementary Fig. 2). These features are typical for *S* = 1/2 transition metal ions in zeolite systems^53–55^ and diagnostic of M–O–Al linkages, demonstrating that under hydration conditions a fraction of the Cu^II^ ions maintains, at least, a partial interaction with the zeolite framework, in agreement with the RT CW-EPR spectrum. At increasing dehydration temperatures, the ^27^Al signals drastically change, evolving from well-defined cross peaks in the hydrated sample to a unique unresolved diffuse signal in the fully dehydrated sample (Fig. 2c, at the bottom and Supplementary Fig. 3). This behaviour corresponds to a continuous increase of the aluminium quadrupolar interaction upon water removal, consistent with previous reports on metal-doped zeolites^56,57^ and quantum mechanical modelling (Supplementary Table 7).

The full set of ^27^Al and ^1^H hfi evaluated through the simulation of HYSCORE and ENDOR spectra (for the simulation of HYSCORE spectra, see Supplementary Figs. 2 and 3) are listed in Table 2.

**Table 2:** Experimental ^1^H and ^27^Al hyperfine coupling components and quadrupolar coupling constants used for the simulations of HYSCORE and ENDOR spectra in Fig. 2.

Nucleus			*a*_*iso*_	*T*_*1*_	*T*_*2*_	*T*_*3*_	*[α, β, γ]*	*e*^*2*^*qQ/h*	*[α’, β’, γ’]*
^1^H	Hydrated	H(1)	−1.5 ± 0.2	−4.0 ± 0.3	−6.0 ± 0.2	10 ± 0.8	[0, 90, 0] ± 10		
		H(2)	0.3 ± 0.2	−3.0 ± 0.2	−4.0 ± 0.3	7.0 ± 0.5	[0, 20, 0] ± 5		
		H(3)	−0.8 ± 0.2	−0.4 ± 0.3	−0.4 ± 0.3	−0.8 ± 0.3	[0, 50, 0] ± 20		
	Part. hydrated	H(4)	−2.2 ± 0.2	−4.0 ± 0.3	−7.0 ± 0.2	11 ± 0.8	[0, 85, 0] ± 5		
^27^Al	Hydrated	Al(1)	−2.3 ± 0.2	−1.0 ± 0.2	−1.0 ± 0.2	2.0 ± 0.4	[0, 0, 0] ± 5	≤4	[0, 20, 0] ± 5
	Dehydrated	Al(2)	−3.0 ± 0.5	2.0 ± 0.2	2.0 ± 0.2	−4.0 ± 0.3	[0, 0, 0] ± 5	11 ± 5	[0, 90, 0] ± 5

All hyperfine interactions are given in units of MHz, while angles are in degrees.

Summarizing, the combination of CW-EPR and hyperfine techniques, provides evidence, in the hydrated sample, for solvated and mobile [Cu(H_2_O)_6_]^2+^ species along with framework interacting species, which attain a partially solvated structure bearing intimate contact with the framework. Upon dehydration, the two species adopt a tetragonal planar coordination through coordinating oxygen donor atoms of the zeolite cage.

### Nature of the Cu–O bonding interaction from ^17^O EPR

**Fig. 3:**
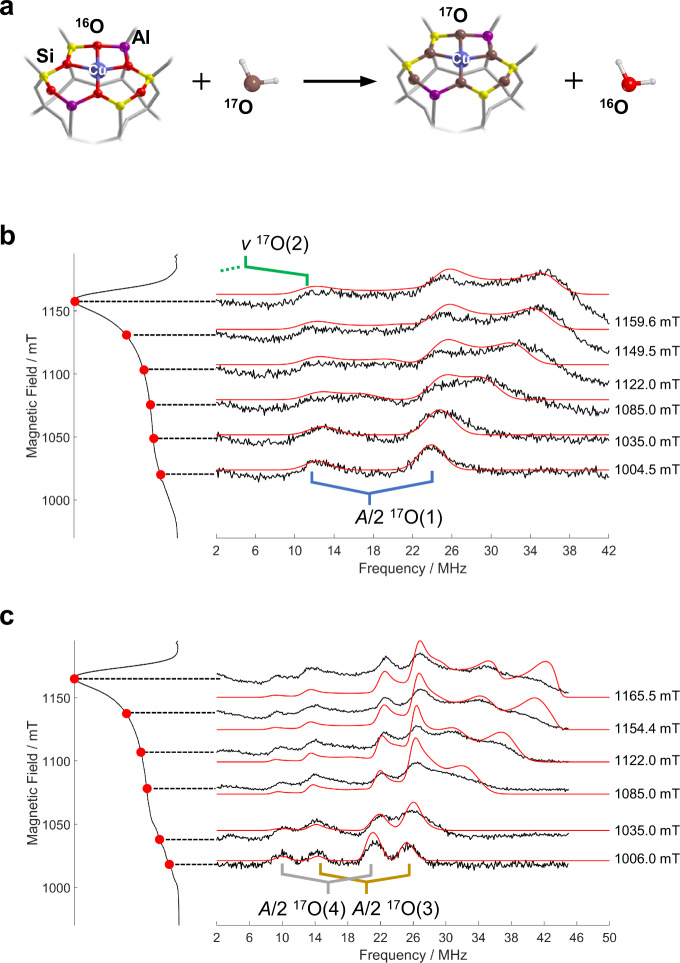
^17^O ENDOR spectra of isotopically enriched Cu-CHA. **a** Schematic representation of the isotopic enrichment of the zeolite framework. **b** Experimental (black) and simulated (red) Q-band ^17^O ENDOR spectra recorded at different magnetic field settings of ^17^O isotopically enriched Cu-CHA fully hydrated with H_2_^17^O. **c** Corresponding experiment performed on a fully dehydrated ^17^O enriched Cu-CHA. The ESE spectra with the corresponding field positions at which the ENDOR spectra were taken are plotted on the left-hand side. All spectra were recorded at 20 K.

Since the SOMO serves as the redox-active orbital in Cu^II^ systems, the covalency of this orbital is crucial for understanding the catalytic properties of Cu based catalysts. The degree of covalency in the ligand–metal bond has far-reaching implications towards reactivity and catalysis as it is the key to activate directional long-range electron transfer pathways^58,59^, enhance catalyst stability^60^ and determine the selective stabilization of intermediate species in redox reactions^61^. Detailed information on the Cu–O bonding interaction can be obtained through the detection of the ^17^O hyperfine interaction, which is a direct reflection of the spin delocalization over the coordinating ligands and a direct probe of the metal-ligand covalent character. To enable the detection of ^17^O hyperfine interactions we isotopically enriched the zeolite framework following protocols previously introduced by some of us^62^. Hydration and dehydration cycles with ^17^O enriched water lead to framework ^17^O incorporation. As reported by some of us^62^ and confirmed by ^17^O NMR studies^22^, under the mild reaction conditions adopted in this work, both Al–O and Si–O bonds undergo ^17^O isotopic exchange. This is schematically shown in Fig. 3a, where the exchangeable sites are highlighted. Orientationally selected ^17^O Davies ENDOR spectra of the hydrated and fully dehydrated zeolite are shown in Fig. 3b and c, respectively. An ENDOR signal represents an NMR absorption which is observed as a change in the echo signal intensity at a fixed resonant magnetic field, **B**_**0**_.

The ENDOR pattern for the *Δ*_m_ = ±1 transitions for ^17^O (*I* = 5/2), are expected to obey the Eq. (1):

1 $${\nu }_{\pm }({m}_{I}\leftrightarrow {m}_{I}+1)=\left|A/2\pm {\nu }_{I}+3P({m}_{I}-1/2)\right|$$

where *A* and *P* are the orientation-dependent hyperfine and quadrupole interaction constants respectively and *ν*_*I*_ = 6.73 MHz is the nuclear Larmor frequency of ^17^O at Q-band^63^. When (2|*ν*_*I*_| < |*A*|) as occurs in our case, the equation describes a pattern consisting of two groups of 2*I* lines each, centered at *A*/2 and separated by 2*ν*_*I*_. Within each group the resonances are separated by 3*P*.

The low field ^17^O ENDOR spectrum of the hydrated zeolite (Fig. 3b) corresponds to a single crystal-like orientation and is characterized by an unresolved set of 2*I* = 5 quadrupole lines separated by 2*ν*_*I*_ and centered at a frequency corresponding to *A*/2 (^17^O(1) in Fig. 3b). The higher-frequency component is more intense, probably due to hyperfine enhancement and/or due to radio frequency (RF) conversion efficiency of the RF coils. A second oxygen (^17^O(2) in Fig. 3b) with an hyperfine coupling of the order of 12 MHz, close to the cancellation regime (2|*ν*_*I*_| ≈ |*A*|), is also present and responsible for the resonance fixed at approximately 12 MHz.

To identify the oxygen ligand containing ^17^O and ascertain the presence of framework coordination, experiments were performed on a ^17^O isotopically enriched Cu-CHA zeolite (Supplementary Fig. 4), subsequently hydrated with normal water. The presence of ^17^O resonances (Supplementary Fig. 5), similar to those reported in Fig. 3b in the ENDOR spectra, demonstrates the presence of an intimate interaction of Cu^II^ species with the zeolite framework under hydrating conditions supporting the assignment based on ^27^Al HYSCORE spectra. By fitting the experimental spectra taken at various resonant magnetic field the full ^17^O interacting tensor for the two families of interacting nuclei was recovered with **A**_O(1)_ = [−36.5 −36.5 −60.5] MHz and **A**_O(2)_ = [−8 −8 −14] MHz whereby a negative sign was assumed, based on the negative nuclear *g* factor of ^17^O and in agreement with the results of DFT calculations, listed in Supplementary Table 6. The observed couplings can be assigned to equatorially (**A**_O(1)_) and axial (**A**_O(2)_) coordinating oxygens, whereby the difference in the hyperfine coupling reflects the small overlap of the oxygen 2p orbitals of the axial ligands with the Cu 3d_x_^2^_−y_^2^ orbital. The spin density on the two coordinating oxygens is estimated to be ρ_O(1)_ = 5.4% and ρ_O(2)_ = 1.1% (Supplementary Note 1 and Supplementary Table 1)^64^.

ENDOR spectra for the dehydrated zeolite (Fig. 3c) provide a unique level of detail on the dehydrated Cu^II^ docking site, giving evidence of two distinct oxygen species coordinated to the Cu^II^ species. At variance with the fully hydrated system, the single crystal-like spectrum recorded at 1006.0 mT is characterized by two doublets separated by 2*ν*_*I*_ and centered at 37 MHz and 30 MHz, respectively. Simulation of the field dependent spectra allowed to extract the full ^17^O **A**-tensors (Table 3 and Supplementary Fig. 6). The larger ^17^O hyperfine couplings imply an increased spin density transfer over the framework oxygen donor atoms with respect to the hydrated system equivalent to ρ_O(3)_ = 8.5% and ρ_O(4)_ = 6.6%. These values correspond to the covalent contribution to the SOMO per O atom. Considering four coordinating oxygen atoms (Fig. 1b and DFT modelling, *vide infra* and Supplementary Table 2), the wave function is composed by a 70% contribution from Cu 3d_x_^2^_-y_^2^ orbital with the remaining 30% being shared among the 2p orbitals of the lattice oxygen ligands.

**Table 3:** Experimental ^17^O hyperfine coupling components and quadrupolar coupling constants used for the simulations of Davies ENDOR spectra in Fig. 4.

State		*a*_*iso*_	*T*_*1*_	*T*_*2*_	*T*_*3*_	*[α, β, γ]*
Hydrated	^17^O(1)	−44.5 ± 0.2	8.0 ± 0.3	8.0 ± 0.2	−16 ± 0.4	[0, 90, 0] ± 2
	^17^O(2)	−10.0 ± 0.2	2.0 ± 0.2	2.0 ± 0.3	−4.0 ± 0.5	[0, 20, 0] ± 2
Dehydrated	^17^O(3)	−51.0 ± 0.4	12.0 ± 0.3	12.0 ± 0.3	−24.0 ± 0.4	[0, 90, 0] ± 2
	^17^O(4)	−41.0 ± 0.5	9.5 ± 0.3	9.5 ± 0.3	−19.0 ± 0.4	[0, 92, 0] ± 2

The hfi parameters satisfy simultaneously all the six field ^17^O ENDOR spectra. All hyperfine interactions are given in units of MHz, while angles are in degrees.

Based on the ENDOR linewidth, we estimate an upper limit of the order of 7 MHz for the ^17^O nuclear quadrupole coupling constant (*e*^*2*^*qQ/h*) for both anhydrous and hydrated conditions. These values are consistent with those reported from ^17^O-NMR studies^21^ for Brønsted acid sites of 6.6 MHz and in line with computed values reported in Supplementary Tables 6 and 7.

The contribution from the different species were properly weighted in the simulation in order to better fit the experimental plot. ^17^O(3) and ^17^O(4) species were considered in 1:1 ratio, whereas ^17^O(1) bestows the 95 % of the simulated signal and ^17^O(2) accounts for the remaining 5%. H(1), ^17^O(1), ^17^O(3) and ^17^O(4) hyperfine tensors are found to be in the same plane (same Euler *β* angle which expresses the orientation of **A**-tensor with respect to **g**-tensor). In the same way, Al(1), H(2) and ^17^O(2) hfi tensors are almost collinear to each other. A summary of the ^17^O spin-Hamiltonian parameters adopted for simulating the ENDOR spectra is given in Table 3.

### The microscopic structure of Cu^II^ in CHA and Al siting: DFT Modelling

**Fig. 4:**
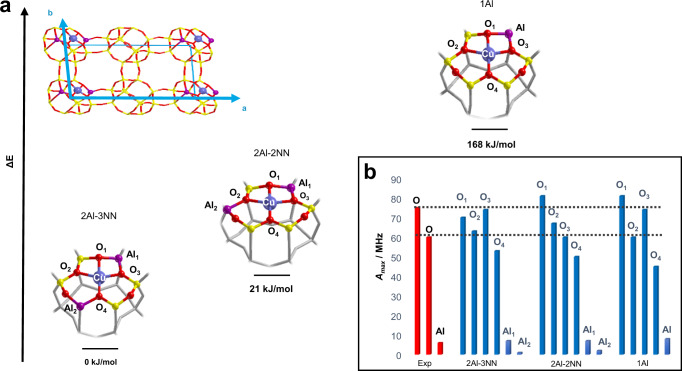
Computed energetics and ^17^O hyperfine couplings of relevant Cu-CHA structures. **a** B3LYP-D3(ABC)/pob-TZVP fully optimized structures of dehydrated Cu-CHA models (Si/Al = 11) by employing three different Al distributions, namely 2Al-3NN, 2Al-3NN and 1Al. The corresponding relative electronic energy per unit cell is shown for each structure. The unit cell (top left of the figure) is oriented according to the lattice vectors reported in cyan. **b** Comparison of experimental (red) and computed (blue) maximum hyperfine coupling values (*A*_max_) for ^17^O and ^27^Al nuclei for the three different Al distributions illustrated in (**a**). The black dashed lines represent the experimental range of *A*_max_ for ^17^O nuclei.

To transpose the spectroscopic findings into atomistic models, DFT calculations were carried out for copper docking sites differing in the Al distribution, namely two Al in two adjacent D6MR, two Al in the same D6MR at second-nearest-neighbour positions and two Al in the same D6MR at third-nearest-neighbour positions (1Al, 2Al-2NN, 2Al-3NN in Fig. 4a). All the aluminium distributions obey the Loewenstein rule avoiding two adjacent sites to be occupied by Al ions^65^.

The relative energy of Cu^II^ at the three sites was computed for two different Si/Al ratios (Si/Al = 11 and Si/Al = 5). The optimized structures all converged to a tetragonal coordination by the lattice oxygen donor atoms (henceforth named $[{{{{{{\rm{Cu}}}}}}}^{{{{{{\rm{II}}}}}}}{\left({{{{{\rm{O}}}}}}-6{{{{{\rm{MR}}}}}}\right)}_{4}]$). The most energetically stable configuration was obtained for 2 Al at third-nearest-neighbour (2Al-3NN) positions in a 6MR unit independent of the Si/Al ratio, in agreement with the previous evidence^3,31^.

For each model, the spin-Hamiltonian parameters were calculated and compared to the experimental data. The computed **g**- and copper **A**-tensors obtained by using the geometry optimized structures are reported in Supplementary Table 4 and are in qualitative agreement with the experimental data, but do not allow to confidently discriminate among the three different possible structures presented in Fig. 4. This is unsurprising as the precise and robust calculation of **g**- and **A**-tensors for Cu^II^ systems is still a great challenge for quantum chemistry methods and only qualitative agreements are usually obtained^66–68^. On the other hand, the prediction of hyperfine couplings for lighter elements (due to negligible spin-orbit coupling and a more accurate determination of the Fermi contact term) is far more reliable^69^. In Fig. 4b the maximum hyperfine coupling values (*A*_max_ = |*a*_iso_ + 2 *T*|) for ^17^O and ^27^Al nuclei is plotted for the three different structures and compared with the experimental values. Examination of the computed values shows that the experimental ^27^Al couplings are quantitatively reproduced, however they are not diagnostic as all models yield very similar values. In particular, in the case of the 2 Al models (2Al-3NN and 2Al-2NN in Fig. 4b) the computed couplings display analogous values (∼7 MHz and ∼1 MHz) irrespectively of the Al location. While the 7 MHz coupling is in agreement with the experimental value of 6 MHz, typical for Cu loaded zeolites^55^, the 1 MHz coupling is too small to be confidently measured, making it impossible to discriminate between 2 Al and 1 Al sites. ^17^O hyperfine couplings prove to be a far more sensitive structural probe. The experimental data (Table 3) point to two sets of ^17^O nuclei characterized by different couplings, whereas four different couplings are computed for all models. However, inspection of Fig. 4b shows that only for 2Al-3NN two classes of alike couplings (O_1_, O_3_ and O_2_, O_4_) can be recognized, with values falling within the experimental range, while 1 Al and 2Al-2NN models feature remarkably different couplings for all four oxygen nuclei (see also the simulation of the ^17^O ENDOR spectra considering the DFT calculated hyperfine couplings in Supplementary Fig. 7).

The differences in the ^17^O hyperfine couplings do not arise from differences in the Cu–O bond lengths (Supplementary Table 2), but rather in slightly different spin density transfer over the coordinating oxygen atoms. This can be traced back to geometric distortions from square planar coordination, resulting in a decreased overlap between the Cu d_x_^2^_−y_^2^ and the O 2p orbitals. A similar effect is observed for [CuCl_4_]^2−^ complexes where the covalent contribution of the ligands to the SOMO is found to increase from D_2d_ to D_4h_ symmetries due to the increasing overlap between the Cu d_x_^2^_−y_^2^ orbital and the Cl 3p orbitals in the two geometries^70^. The three different sites considered in this work feature very similar Cu–O distances but slightly different geometric distortions, leading to asymmetries in the spin delocalization, characteristic for the different sites. Crucially, asymmetries in the spin delocalization are of outmost relevance as they report on the covalent character of each ligand–metal bond, which ultimately design preferential electron transfer pathways^58,59^.

Overall, this analysis shows that not only structure 2Al-3NN is the most energetically favoured, but it is also the only one for which a satisfactory agreement between computed and experimental spin-Hamiltonian parameters is obtained. In turn, this allows to confidently conclude that 2Al-3NN sites are those dominantly populated by Cu^II^ species under the experimental conditions.

**Fig. 5:**
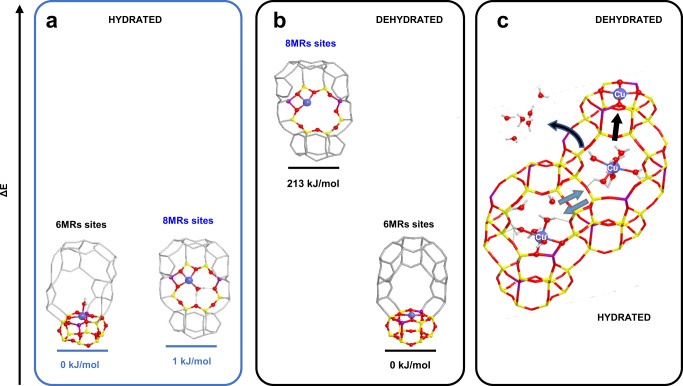
Computed energies of hydrated and dehydrated Cu-CHA models. **a** Hydrated and **b** dehydrated Cu^II^ species in 6MRs and 8MRs sites with the corresponding relative electronic energy per unit cell. **c** Picture of the Cu^II^ dynamics inside the CHA framework due to the presence of water molecules. Some relevant atoms are represented by balls-and-sticks (colour code: medium blue Cu, yellow Si, violet Al, red O, white H).

A similar analysis on the hydrated system, yields a different scenario for the Cu^II^ docking and allows the assessment of migration pathways induced by hydration/dehydration processes. Based on experimental UV-vis, IR and XAS spectra of Cu-CHA in hydrated state at room temperature, previous studies have assumed the presence of two types of Cu^II^ complexes, regardless the copper loading and the composition of the zeolite: the former are divalent complexes charge-compensated by a pair of Al atoms (like [Cu(H_2_O)_6_]^2+^ or [Cu^II^(O-8MR)_6-n_(H_2_O)_n_]); the latter are monovalent complexes charge-compensated by one Al atom (e.g. [Cu^II^(OH)(H_2_O)_5_] or [Cu^II^(OH)(O-8MR)_5−n_(H_2_O)_n_])^31,39^. However, the discrimination among such hydrated complexes is hampered by the resolution of the experimental techniques mentioned above. To shed light on the nature of the hydrated species, we first assessed the relative stability of Cu^II^ at the different sites as a function of hydration by considering the effect of a single water molecule coordinated to Cu^II^ ions hosted in 6MR and 8MR sites, as illustrated in Fig. 5a, b.

When we consider a dehydrated Cu^II^ ion, the 6MR site is far more stable than the 8MR because of the stronger binding to the four framework oxygen atoms in a tetragonal geometry. Energetic considerations suggest thus such a location is the preferred one for the dehydrated material, in agreement with several experimental and theoretical studies^35,37,71^. However, the adsorption of water molecules completely alters the relative stabilities: with one water ligand, the stability of both sites becomes similar (Fig. 5a). Further addition of H_2_O molecules increases the energy gap between the different cage locations pointing out that, in hydrated and partially hydrated conditions (both experimentally observed), 8MR sites are more stable than 6MR, in agreement with the findings of Kerkeni et al.^72^. Details on such results are reported in Supplementary Fig. 8.

**Fig. 6:**
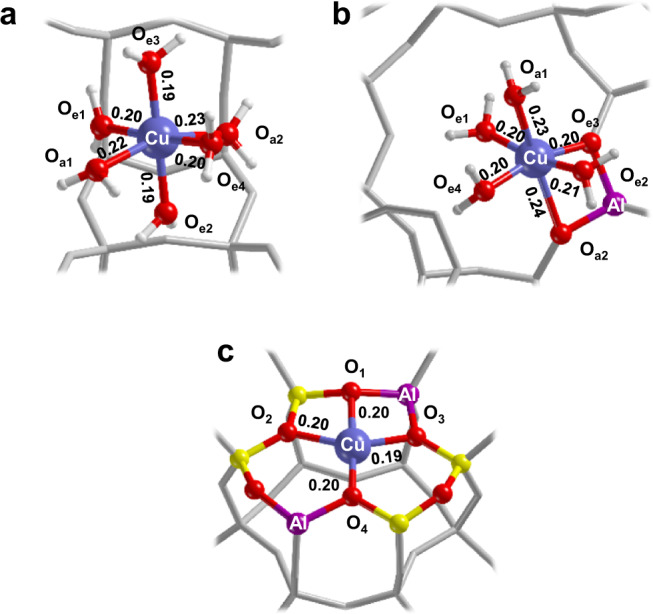
Atomistic structures of the Cu^II^ interfacial complexes in CHA with composition Si/Al = 15 and Cu/Al = 0.005. **a** $[{{{{{{\rm{Cu}}}}}}}^{{{{{{\rm{II}}}}}}}{({{{{{{\rm{H}}}}}}}_{2}{{{{{\rm{O}}}}}})}_{6}]{{{{{\rm{@CHA}}}}}}$ in the Chabazite’s largest cage, **b** $[{{{{{{\rm{Cu}}}}}}}^{{{{{{\rm{II}}}}}}}{({{{{{{\rm{H}}}}}}}_{2}{{{{{\rm{O}}}}}})}_{4}{({{{{{\rm{O}}}}}}-8{{{{{\rm{MR}}}}}})}_{2}]$ complex and **c** $[{{{{{{\rm{Cu}}}}}}}^{{{{{{\rm{II}}}}}}}{\left({{{{{\rm{O}}}}}}-6{{{{{\rm{MR}}}}}}\right)}_{4}]$ complex at the 2Al-3NN site. Cu–O bond lengths are indicated in nm. The label of relevant nuclei is reported whereas Si and H atoms are shown in yellow and white, respectively. The remaining framework atoms are represented by grey sticks.

The spin Hamiltonian parameters for the hydrated structures shown in Fig. 6a, b and hydrated [CuOH]^+^ species (Supplementary Fig. 10) were computed. The ^1^H hyperfine couplings for the [Cu(H_2_O)_6_]^2+^ (Fig. 6a) and interfacial Cu species (Fig. 6b) are in quantitative agreement with the experimental values and characteristic for axially (small hyperfine coupling) and equatorially (large hyperfine coupling) water bound protons. On the other hand, the computed ^1^H hyperfine coupling of the hydroxyl proton of [CuOH]^+^—about 20 MHz (Supplementary Table 8 and Supplementary Note 3)—is exceedingly large and allows to discard this structure, consistently with the small Cu/Al ratio. This is also in agreement with the relatively small reduction of the sample upon dehydration compared to similar systems with higher Cu loadings and concur to confirm that 2 Al sites are preferentially populated, consistently with energetic considerations. Computed oxygen hyperfine couplings for these structures (Supplementary Table 6) reproduce the experimental values showing a set of large *a*_*iso*_ couplings (−45 MHz and −55 MHz) related to equatorially coordinated oxygen ligands and a set of small values (−8 MHz and −6 MHz) associated to axially coordinated oxygen ligands.

In summary, two Cu^II^ complexes are identified under hydrating conditions, $[{{{{{{\rm{Cu}}}}}}}^{{{{{{\rm{II}}}}}}}{({{{{{{\rm{H}}}}}}}_{2}{{{{{\rm{O}}}}}})}_{6}]{{{{{\rm{@CHA}}}}}}$ (Fig. 6a) and an interfacial complex $[{{{{{{\rm{Cu}}}}}}}^{{{{{{\rm{II}}}}}}}{({{{{{{\rm{H}}}}}}}_{2}{{{{{\rm{O}}}}}})}_{4}{({{{{{\rm{O}}}}}}-8{{{{{\rm{MR}}}}}})}_{2}]$ (Fig. 6b), whereby with the notation @CHA we indicate the hexaaquacopper complex encapsulated in the Chabazite’s largest cage. The CHA cage has a diameter of 1.2 nm ensuring the possibility of free tumbling of the water complex at room temperature and explaining the motionally averaged spectrum observed at RT. This mobile species coexists along with the $[{{{{{{\rm{Cu}}}}}}}^{{{{{{\rm{II}}}}}}}{({{{{{{\rm{H}}}}}}}_{2}{{{{{\rm{O}}}}}})}_{4}{({{{{{\rm{O}}}}}}-8{{{{{\rm{MR}}}}}})}_{2}]$ complex (Eq. (2) and Fig. 5c), where two water ligands are substituted by two oxygen donor atoms belonging to the 8MR site as demonstrated by ^17^O ENDOR spectra recorded on the ^17^O-exchanged zeolite in the presence of H_2_^16^O (Supplementary Fig. 5).

2 $$[{{{{{\rm{Cu}}}}}}^{{{{{\rm{II}}}}}}({{{{{\rm{H}}}}}}_{2}{{{{{\rm{O}}}}}})_{4}({{{{{\rm{O}}}}}}-8{{{{{\rm{MR}}}}}})_{2}]\rightleftarrows [{{{{{\rm{Cu}}}}}}^{{{{{\rm{II}}}}}}({{{{{\rm{H}}}}}}_{2}{{{{{\rm{O}}}}}})_{6}]{{{{{\rm{@CHA}}}}}}$$

The hydration–dehydration processes in Cu-CHA can, therefore, be described in terms of a dynamic conversion (Eqs. (3) and (4)) between water solvated species and an interfacial complex $[{{{{{\rm{Cu}}}}}}^{{{{{\rm{II}}}}}}({{{{{\rm{O}}}}}}-6{{{{{\rm{MR}}}}}})_{4}]$, whereby the 6MR cage behaves as a tetradentate ligand as shown in Fig. 6c.

3 $$\left[{{{{{\rm{Cu}}}}}}^{{{{{\rm{II}}}}}}{({{{{{\rm{H}}}}}}_{2}{{{{{\rm{O}}}}}})}_{4}{({{{{{\rm{O}}}}}}-8{{{{{\rm{MR}}}}}})}_{2}\right]^{{\mathop{\longrightarrow }\limits^{{-{{{{{\rm{H}}}}}}_{2}{{{{{\rm{O}}}}}}}}}}_{\mathop{\longleftarrow }\limits_{{+{{{{{\rm{H}}}}}}_{2}{{{{{\rm{O}}}}}}}}}\left[{{{{{\rm{Cu}}}}}}^{{{{{\rm{II}}}}}}{({{{{{\rm{O}}}}}}-6{{{{{\rm{MR}}}}}})}_{4}\right]$$

4 $$\left[{{{{{\rm{Cu}}}}}}^{{{{{\rm{II}}}}}}({{{{{\rm{H}}}}}}_{2}{{{{{\rm{O}}}}}})_{6}\right]{{{{{\rm{@CHA}}}}}}^{\mathop{\longrightarrow }\limits^{{-}{{{{{\rm{H}}}}}}_{2}{{{{{\rm{O}}}}}}}}_{\mathop{\longleftarrow }\limits_{{+}{{{{{\rm{H}}}}}}_{2}{{{{{\rm{O}}}}}}}}\left[{{{{{\rm{Cu}}}}}}^{{{{{\rm{II}}}}}}({{{{{\rm{O}}}}}}-6{{{{{\rm{MR}}}}}})_{4}\right]$$

The ${[{{{{{{\rm{Cu}}}}}}}^{{{{{{\rm{II}}}}}}}\left({{{{{\rm{O}}}}}}-6{{{{{\rm{MR}}}}}}\right)_{4}]}$ interfacial complexes feature a SOMO with predominant metal d_x_^2^_−y_^2^ character and a covalent contribution of about 30% from the coordinating framework oxygen donor atoms. Importantly, DFT calculations demonstrate that the spin density distribution over the coordinating oxygens and the corresponding ^17^O hyperfine couplings are significantly dependent upon the Al distribution in the 6MR site, allowing to discriminate between different Al locations. In particular we find that for the low Cu loading considered in this work, the 2Al-3NN site, characterized by 2 Al atoms separated by two Si, not only has the lowest energy but also provides the better agreement between calculated and experimental ^17^O hyperfine coupling constants. The new methodology and associated new knowledge that emerges from this study will need to be applied systematically to other framework topologies and comparison will need to be set to catalysts characterized by different Si/Al and Cu/Al ratios. This systematic approach will form the basis to correlate catalytic performances to the Cu (and in general paramagnetic metal species) location at specific zeolite sites. Moreover, while providing a strategy to optimizing catalyst compositions (Al distribution, Cu loading, etc.) the detection of ^17^O hyperfine couplings provides an effective means to selectively probe the framework lability at open-shell metal centres in zeolites. Most importantly, the exquisite sensitivity of such couplings enables to account for minute structural differences related to the Al distribution and identify the Al siting in the most stable Cu^II^ coordination, a long-standing issue in the field.

## Methods

### Sample preparation and treatment

Na-CHA was prepared using N,N,N-trimethyladamantanammonium hydroxide (TMAdaOH) as template following the procedure published in the patent literature^73^. The resulting synthesis gel was transferred to a Teflon-lined steel autoclave and heated to 140 °C for 6 days. The product was recovered by centrifugation, washed several times with deionized water, dried overnight at 75 °C and calcined in air at 550 °C for 8 h to remove the TMAdaOH. The resulting zeolite was a pure Na-CHA (Si/Al = 15) without FAU impurities.

Prior to the copper exchange, the protonated form of the zeolite was obtained by liquid ion exchange with a 10% solution of ammonia nitrate, drying in the oven (80 °C) overnight and heating at 550 °C for 3 h to remove the ammonia residues from the framework. Therefore, the doping of Cu^II^ cations inside the H-CHA sample was performed by following the ion exchange procedure described by Kevan et al.^18^. According to this method, approximately 1 Cu^II^ cation is incorporated per each 100 unit cells ensuring a very low copper content and a good dilution of the paramagnetic centers, which is fundamental for the application of advanced EPR techniques and allows titrating the most energetically favourable sites. The elemental percentage composition of Cu-CHA sample, determined by ICP-AES analysis, is the following: 43.70 wt% of Si, 2.72 wt% of Al and 0.03 wt% for Cu. No Na residues were detected by ICP-OES. Hence, the Cu/Al ratio is 0.005. The absolute quantification of Cu^II^ in the fully hydrated sample was estimated to be of the same order of magnitude of the total Cu content determined by ICP-AES. The SpinCount package of Bruker Xenon Software was employed to carry out the EPR quantification.

The dehydration of the zeolite was carried out at several temperatures under dynamic vacuum (final pressure <10^−4^ mbar) for a maximum time of 2 h.

The sample was isotopically enriched by exposing the dehydrated powder of Cu-CHA to three consecutive hydration and dehydration cycles in presence of vapours of H_2_^17^O (86% isotopic enrichment supplied by Icon Services New Jersey) at 120 °C for 2 h: this method was proved to be extremely efficient in the ^17^O enrichment of zeolites without causing any dealumination of the framework^62^. At the end of the process, a ^17^O enriched Cu-CHA sample hydrated with H_2_^17^O was obtained and, after a final dehydration step, the isotopically labelled water was removed from the framework. Finally, the dehydrated ^17^O labelled sample was rehydrated with H_2_^16^O by merely exposing the solid to air for 24 h in order to further prove the framework substitution of ^16^O with ^17^O.

### EPR measurements

The X-band (microwave frequency of 9.42 GHz) continuous-wave (CW)-EPR spectra were detected at 298 K and 77 K on a Bruker EMXmicro spectrometer. A modulation amplitude, modulation frequency and microwave power of 10 G, 100 kHz and 2 mW were used, respectively. Pulse EPR experiments were performed at 4.5 K and 20 K with X-band (microwave frequency of 9.75 GHz) and Q-band (microwave frequency of 33.8 GHz) Bruker ELEXYS 580 EPR spectrometers equipped with helium gas-flow cryostat from Oxford Inc. The magnetic field was measured with a Bruker ER035M NMR gaussmeter.

The electron-spin-echo (ESE) detected EPR spectra were recorded with the pulse sequence $\frac{\pi }{2}-\tau -\pi -\tau -{echo}$. The pulse lengths ${t}_{\pi /2}=16$ ns and ${t}_{\pi }=32$ ns, a $\tau$ value of 200 ns and a shot repetition rate of 3.55 kHz.

X-band hyperfine sublevel correlation (HYSCORE)^74^ experiments were performed with the pulse sequence $\pi /2-\tau -\pi /2-{t}_{1}-\pi -{t}_{2}-\pi /2-\tau -{echo}$, applying a four-step phase cycle for deleting unwanted echoes. Pulse lengths of ${t}_{\pi /2}=16$ ns and ${t}_{\pi }=32$ ns and a shot repetition rate of 1.77 kHz were used. The increment of the time intervals ${t}_{1}$ and ${t}_{2}$ was 16 ns, starting from 80 to 2704 ns giving a data matrix of $170\times 170$; the pulse delay $\tau$ value was set to 104 ns. The time traces of HYSCORE spectra were baseline corrected with a third-order polynomial, apodized with a hamming window and zero-filled to 2048 points. After 2D Fourier transformation, the absolute-value spectra were calculated.

Q-band electron nuclear double resonance (ENDOR) measurements were carried out at 15 K and 20 K by employing the Davies pulse sequence^75^ ($\pi -{RF}-\pi /2-\tau -\pi -\tau -{echo}$). For ^17^O nuclei, strongly coupled to Cu^II^ cations, the pulse lengths used were all set to ${t}_{\pi /2}=16$ ns and ${t}_{\pi }=32$ ns. On the other hand, for ^1^H and ^27^Al nuclei weakly coupled to the unpaired electron, the first $\pi$-pulse was directed along the <x> direction with a length of 50 μs in order to selectively excite transitions belonging to nuclei having small hyperfine interactions. The ${RF}$ pulse length was set to $14$ μs and a resolution of 440 points was adopted. Further experimental settings are provided in the figure captions.

All the EPR spectra were simulated by using the Easyspin package (version 6.0.0 dev 26)^76^.

### Periodic and cluster modelling

In the present work, Chabazite structure was modelled by employing periodic boundary conditions which better describe the crystalline environment of the zeolite with respect to cluster approaches. Firstly, a purely siliceous chabazite (space group $R\bar{3}m$) composed by a rhombohedral lattice with 12 tetrahedral (T) sites per unit cell was considered. Therefore, two Si atoms were replaced by two Al atoms generating a model with a Si/Al ratio equal to 5. The excess of negative charge is exactly compensated by one Cu^II^ cations per unit cell. Structures with different distributions and amount (Si/Al ratio of 5 and 11) of aluminium atoms were fully optimized in P1 space group without any symmetry constrain in order to find the most stable Al configuration. Additional models in which the double positive charge of copper ions is counterbalanced by one Al substitution and a OH^−^ group (Si/Al ratio of 11) are also reported in Supplementary Note 3. Hydrated copper species incorporated inside the CHA framework were simulated by adding water molecules to the previously optimized structures or by inserting a Cu molecular complex inside the CHA cavity and reoptimizing the whole adduct.

The periodic DFT study has been complemented with molecular cluster calculations in order to estimate the **g**-tensor of Cu^II^ and the relative orientations of the ^17^Al, ^1^H and ^17^O hyperfine interactions with respect to the g-frame. Cluster models were cut out from the corresponding optimized periodic structures. The dangling bonds were saturated with hydrogen atoms oriented along the broken bonds to keeping the local environment as in the optimized periodic models. Thus, no further geometry optimization of the cluster models was performed: the **g**-tensor was computed maintaining the same atomic coordinates as the ones in the relaxed periodic structures. The net charge on the clusters was set to 0 in a doublet spin state, except for the hexaaquacopper complex for which a charge of +2 was assessed.

### Computational details

Periodic geometry optimizations were carried out by using the distributed parallel version of CRYSTAL17 code (PCRYSTAL)^77^ in the frame of Density Functional Theory (DFT) adopting the hybrid B3LYP method, Becke’s three parameters exchange functional and the correlation functional from Lee, Yang and Parr^78,79^. The semi-empirical dispersion corrections for the vdW interactions were employed by using the Grimme approach in the so-called DFT-D3 method in conjunction with a three-body correction^80,81^. The pob-TZVP basis set^82^ was used for all the elements during the geometry relaxation of both atomic coordinates and cell parameters. H and O atoms from extra-lattice water molecules or hydroxyl group were treated with Ahlrichs VTZP basis set^83^. For the prediction of ^27^Al, ^1^H and ^17^O hfi, single point calculations performed on the optimized structures were carried out by employing the aug-cc-pVTZ-J^84^ basis set for Al atoms, and the EPR-III^85^ for H and O atoms at B3LYP-D3. The PBE0^86^-D3 exchange-correlation functional was also assessed for the computation of hyperfine coupling constants of aluminium, hydrogen and oxygen atoms; since the results are very similar to the B3LYP ones, they will not be commented in the following sections. Primitive Gaussians with exponents lower than 0.06 were removed in order to avoid linear dependency in the self-consistent cycle (SCF). The other elements were treated with the same basis sets used for geometry optimizations.

A default pruned grid built according to the Gauss-Legendre quadrature and Lebedev schemes having 75 radial points and a maximum number of 974 angular points in regions relevant for chemical bonding has been adopted. The accuracy of the calculation of the two electron integrals in the Coulomb and exchange series was controlled by imposing truncation criteria set at the values of 10^−7^ except for the pseudo-overlap of the HF exchange series which was fixed to 10^−25^. A shrink factor equals to 6 was used to diagonalize the Hamiltonian matrix in at least 112 k-points of the first Brillouin zone. The default value of mixing (30%) of the Kohn-Sham (KS) matrix at a cycle with the previous one was adopted. The threshold in energy variation of SCF cycles was set equal to 10^−8^ Hartree for both geometry relaxation and magnetic properties evaluation. The number of unpaired electrons in the unit cell of the periodic models was not locked to one in order to leave the SCF procedure to freely converge to a doublet spin state of the system wavefunction.

The EPR/NMR module of the ORCA (v4.2.1) code^87^ was then exploited to compute the **g**-tensor and the Cu hfi on molecular cluster models properly extracted from the optimized periodic structures. The complete mean-field spin-orbit operator (SOMF)^88^ was used for treating the spin-orbit coupling (SOC) which is not negligible for Cu^II^ cations and accounts for a consistent amount of the copper hyperfine **A**-tensor^67,89^. The potential was constructed to include one-electron terms, compute the Coulomb term in a semi-numeric way, incorporate exchange via one-center exact integrals including the spin-other orbit interaction, and include local DFT correlation (SOCFlags 1,2,3,1 in ORCA). The def2-TZVP basis sets^90^ was employed for Si, Al, H and O atoms and the CP(PPP)^91^ for Cu atom. The double-hybrid density functional theory (DHDFT) B2PLYP-D3 method^92^ as implemented in ORCA was adopted for the calculations in combination with the resolution of identity (RI) by using the AutoAux keyword to automatically build the auxiliary basis set. B2PLYP functional is composed by B88 exchange combined with the LYP correlation functional through two empirical parameters controlling the amount of Hartree-Fock (HF) exchange (53% of exact HF exchange) and second order perturbation theory (PT2) correlation. Only the Fermi contact (A^FC^) and the dipolar component (A^SD^) of the hyperfine coupling constant can be computed at MP2 level of theory; the spin-orbit contribution (A^SO^) used was determined at SCF level. In order to compare its performance, the results obtained by B2PLYP-D3 were compared with other exchange-correlation functionals and collected in Supplementary Note 2 and Supplementary Fig. 9.

The SCF convergence criteria were raised up to 10^−8^ Hartree. An integration grid composed by 770 radial points in agreement with the Lebedev scheme was chosen for all the atoms. Regarding the DHDFT method, the core electrons were not frozen in order to allow the computations of the analytical derivatives at MP2 level of theory.

## Supplementary information

Supplementary Information

## Data Availability

The data that support the findings of this study are available in Zenodo with the identifier 10.5281/zenodo.4953846. Ref. ^93^.
