# Supplementary material for: 17O-EPR determination of the structure and dynamics of copper single-metal sites in zeolites
Source: Nat Commun. 2021 Jul 30;12:4638. doi: 10.1038/s41467-021-24935-7 (PMC8324863; doi:10.1038/s41467-021-24935-7)
Supplement: Supplementary file 1 — Supplementary Information [file 41467_2021_24935_MOESM1_ESM.pdf]

^{17}O-EPR determination of the structure and dynamics of copper single-metal sites in zeolites

Paolo Cleto Bruzzese,[1,2] Enrico Salvadori,[2] Stefan Jäger,[3] Martin Hartmann,[3] Bartolomeo Civalleri,[2] Andreas Pöppl[1] and Mario Chiesa[2]**

[1]Felix Bloch Institute for Solid State Physics, Universität Leipzig, Linnéstr. 5, 04103 Leipzig, Germany

[2] Dipartimento di Chimica, Università di Torino and NIS Centre, via P. Giuria 7, 10125, Torino, Italy

[3] Erlangen Center for Interface Research and Catalysis (ECRC), Egerlandstr. 3, 91058 Erlangen, Germany

Corresponding authors: mario.chiesa@unito.it poeppl@physik.uni-leipzig.de

Supplementary Information

Table of Contents

Supplementary Figure 1 1
Supplementary Figure 2 2
Supplementary Figure 3 2
Supplementary Figure 4 3
Supplementary Figure 5 3
Supplementary Figure 6 4
Supplementary Figure 7 5
Supplementary Table 1 6
Supplementary Table 2 6
Supplementary Figure 8 7
Supplementary Figure 9 8
Supplementary Table 3 8
Supplementary Table 4 9
Supplementary Table 5 9
Supplementary Table 6 10
Supplementary Table 7 11
Supplementary Figure 10 12
Supplementary Table 8 13
Supplementary Note 1 13
Supplementary Note 2 13
Supplementary Note 3 14
Supplementary References 15

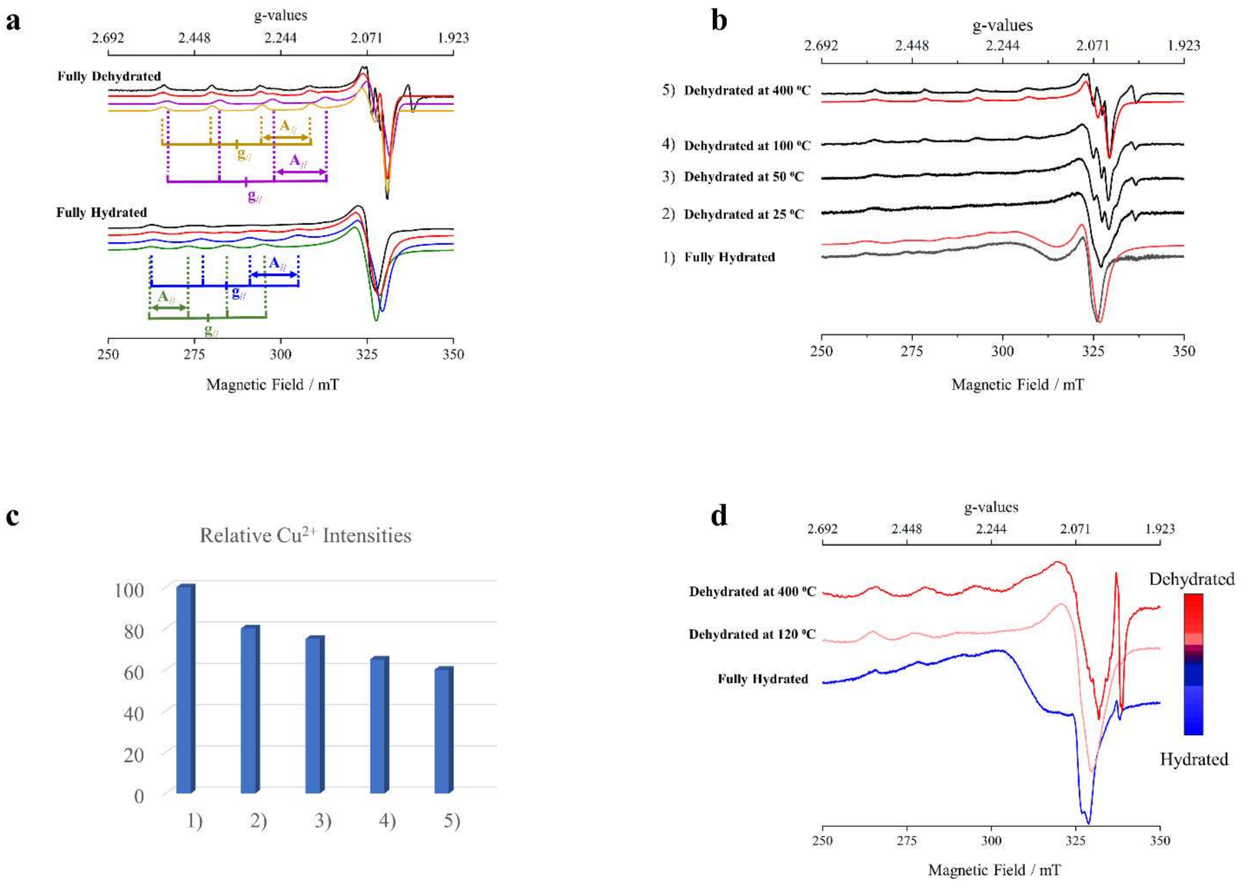

Supplementary Figure 1. X-band CW-EPR spectra of Cu^{II} in CHA. **a** Experimental (black) and simulated (red) X-band CW-EPR spectra recorded at 77 K of fully hydrated and dehydrated Cu-CHA. The contribution of each individual species (A - D in Table 1) is shown in blue, green, gold and violet for A, B, C and D, respectively. **b** Experimental (black) X-band CW-EPR spectra recorded at room temperature at different dehydration stages. The simulations of the spectra of the fully hydrated and dehydrated samples are shown in red. The spectra of the dehydrated sample at RT and 77 K are virtually identical. The simulation of the RT EPR spectrum of the hydrated sample was performed by including a motionally averaged component obtained by imposing a rotational correlation time τ=10^{-11} s to the spin-Hamiltonian parameters determined from the rigid-limit spectrum (Figure S1a). **c** Relative intensity of Cu^{II} signal (%) as determined by double integration of the first derivative spectra. The intensity of the fully hydrated sample was set to 100 %. **d** X-band CW-EPR spectra recorded at room temperature of fully hydrated with $H_2{}^{17}O$ Cu-CHA and dehydrated at increasing temperatures. The line broadening with respect to the corresponding spectra recorded on the $H_2{}^{16}O$ sample is due to the presence of the ^{17}O isotope (I=5/2).

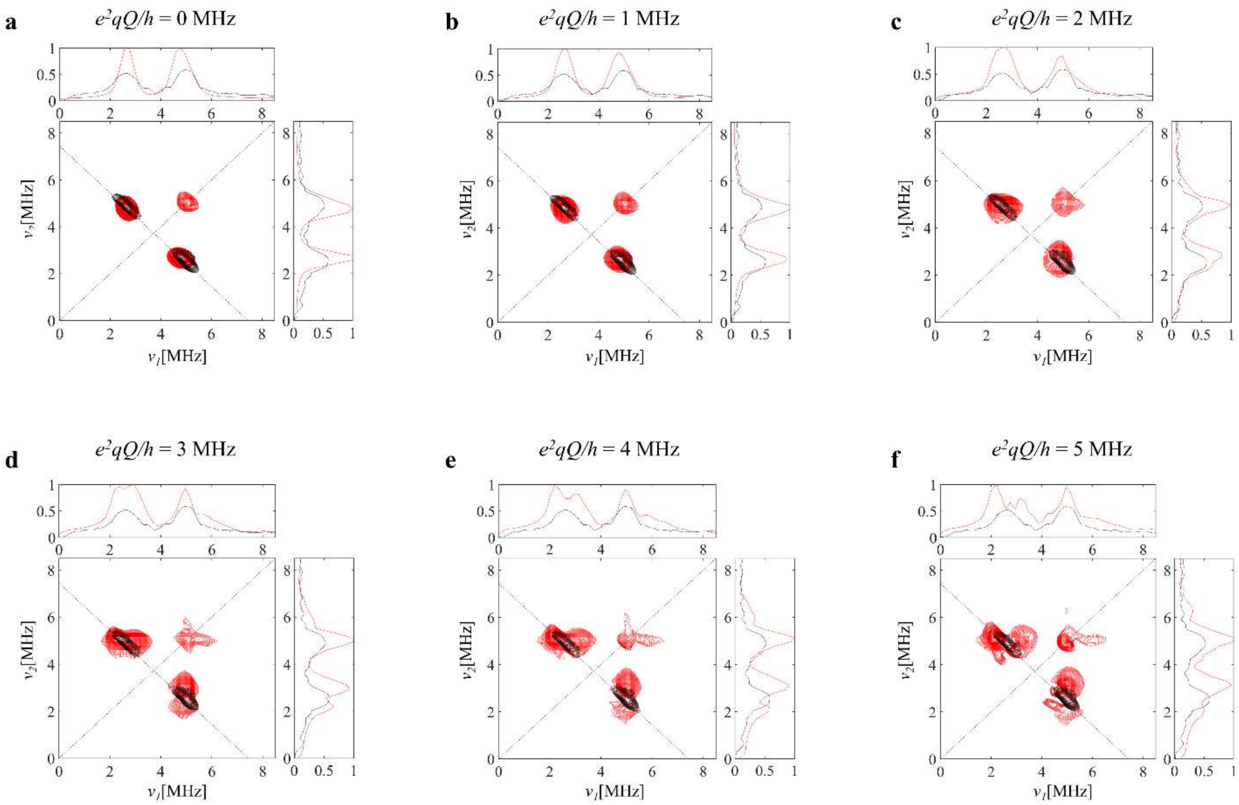

Supplementary Figure 2. Evaluation of the effect of the nuclear quadrupole interaction in X-band ^{27}Al HYSCORE experiments (in black) in the fresh Cu-CHA sample. A e^2qQ/h value of **a** 0 MHz, **b** 1 MHz, **c** 2 MHz, **d** 3 MHz, **e** 4 MHz, **f** 5 MHz was used with [α', β', γ'] angles equal to [0, 20, 0] degrees. In the simulations (in red) the ^{27}Al **A**-tensor and its relative orientation with respect to **g**-tensor employed are the same as reported in Table 2.

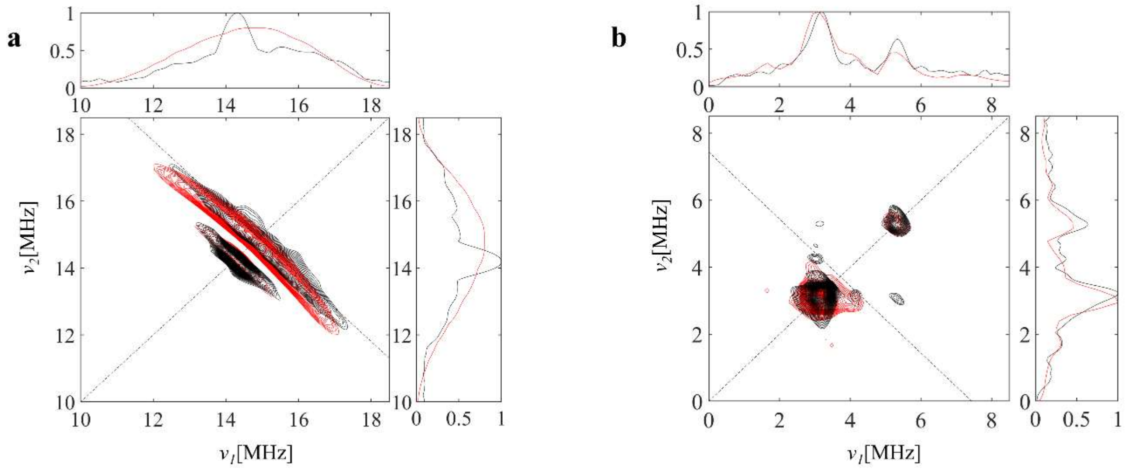

Supplementary Figure 3. a Simulation (in red) of the ^{1}H HYSCORE spectrum (in black) of the fully hydrated Cu-CHA sample. **b** Simulation (in red) of the ^{27}Al HYSCORE spectrum (in black) of the fully dehydrated Cu-CHA sample. Due to the fact that it was impossible to record ^{27}Al HYSCORE spectrum at the $g_{//}$ position because of the low S/N ratio, it is not possible to obtain a univocal set of parameters. A representative simulation obtained using the spin-Hamiltonian parameters reported in

Table 2 is shown which qualitatively reproduces the experimental features. The parameters are consistent with data reported for other Cu-doped zeolites and with the computed values.

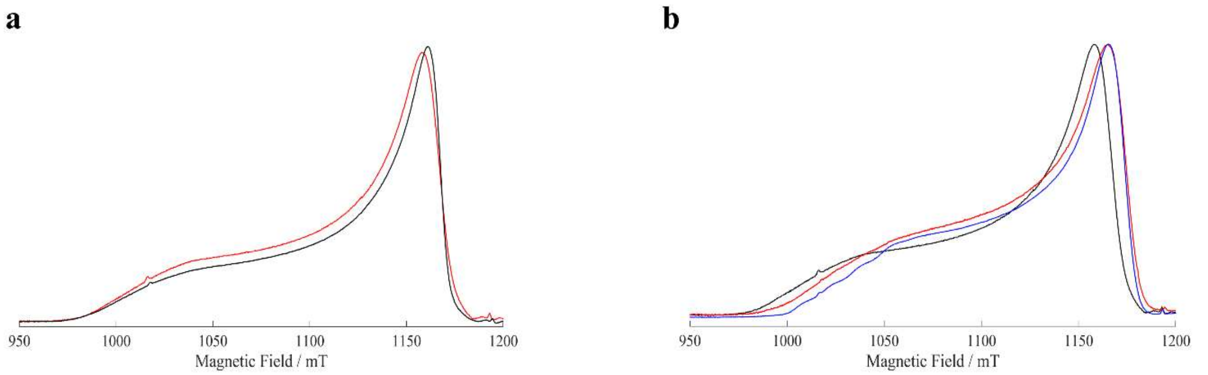

Supplementary Figure 4. a Q-band electron-spin-echo (ESE) detected EPR spectra of Cu-CHA hydrated with $H_2{}^{16}O$ (black line) and with $H_2{}^{17}O$ (red line). The broadening of the spectral line after the isotopic enrichment is due to the intimately interaction of Cu^{II} species with a heavier nucleus (^{17}O instead of ^{16}O). **b** Q-band ESE detected EPR spectra of the isotopically enriched Cu-CHA sample at three different hydration level: fully hydrated (in black), partially hydrated (in red) and completely dehydrated (in blue). The slight shift of the *g*-values with the hydration levels reflects the change of Cu^{II} speciation.

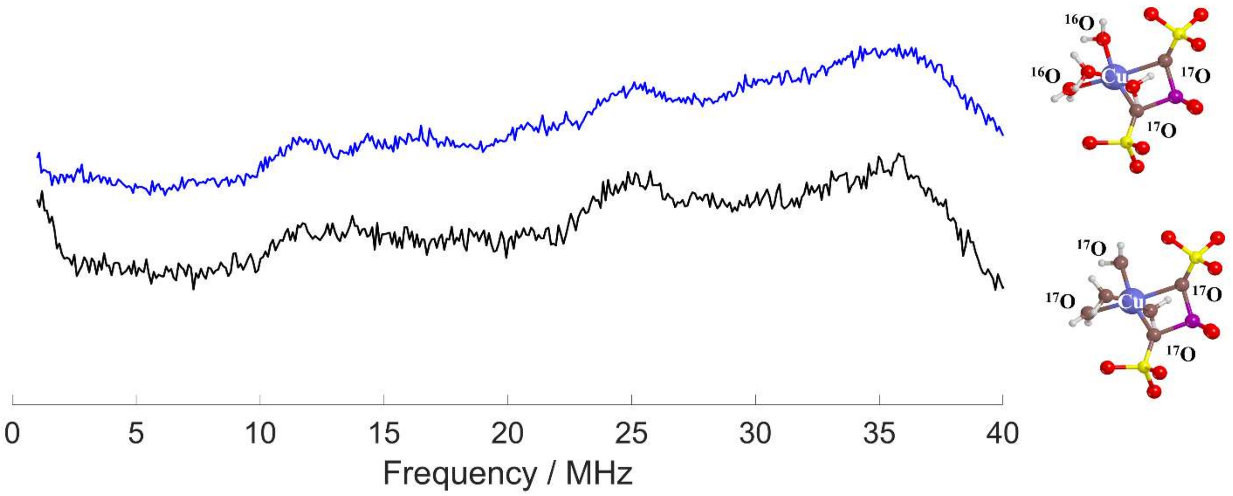

Supplementary Figure 5. ^{17}O Davies ENDOR spectra recorded at the maximum of the echo intensity. The black spectrum refers to Cu-CHA isotopically enriched sample hydrated with $H_2{}^{17}O$

whereas the blue one corresponds to Cu-CHA sample hydrated with normal water after the isotopic enrichment. The corresponding pictorial representation of the Cu site is reported on the right of each spectrum. The similarity of the two spectra demonstrates that Cu^{II} retains a direct linkage with the framework under hydrating conditions and that the water and framework oxygen equatorial ligands display a similar degree of spin density, in accord with the computed data (see O_{e1} and O_{e3} in Table S6). 64 scans were averaged for the ^{16}O solvating water against 25 scans for the ^{17}O solvating water.

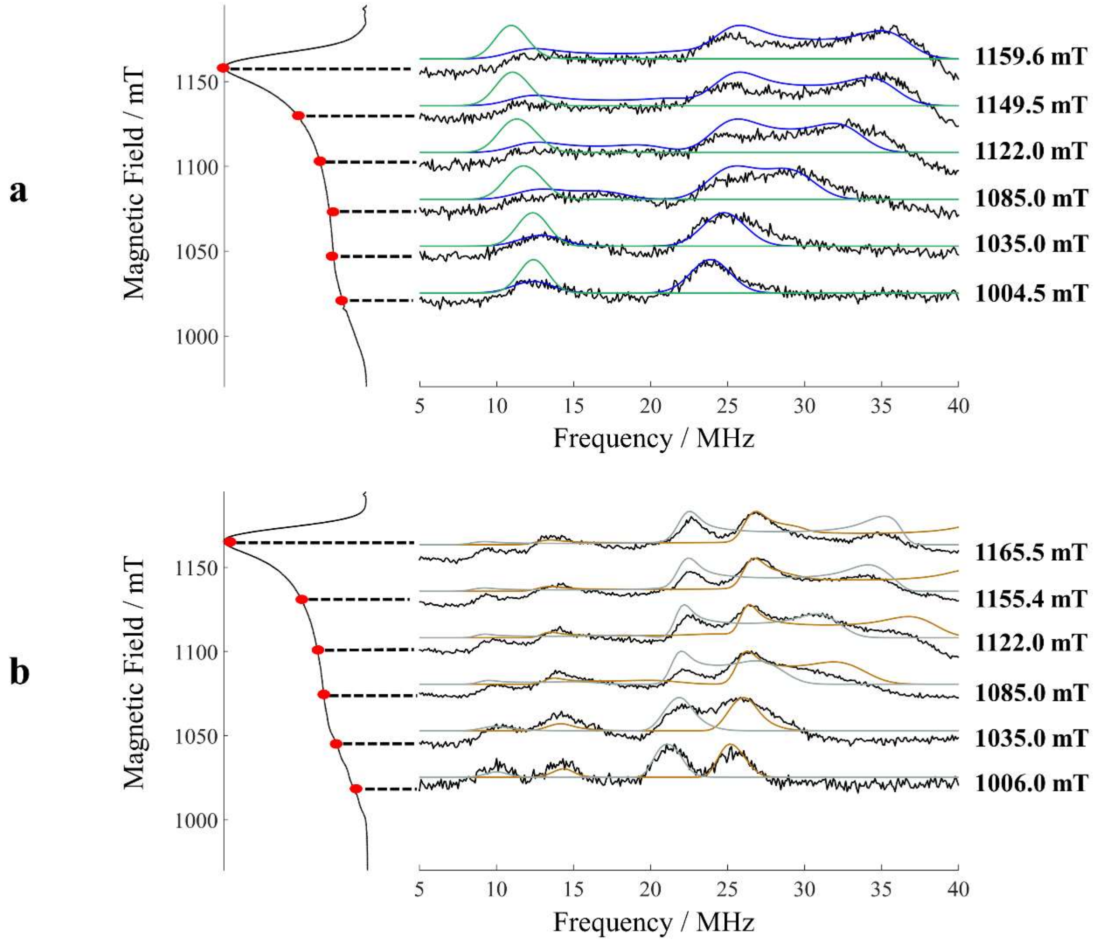

Supplementary Figure 6. Q-band ^{17}O ENDOR spectra simulation highlighting the contribution of each ^{17}O species. The black lines are the experimental spectra. The ESE spectrum with the corresponding field position sampled are plotted on the left. **a** Fully hydrated with $H_2{}^{17}O$. The blue lines represent the simulation obtained with the spin-Hamiltonian of $^{17}O(1)$, the green ones of $^{17}O(2)$. **b** Dehydrated after ^{17}O enrichment. The brown lines represent the simulation obtained with the spin-Hamiltonian of $^{17}O(3)$, the gray ones of $^{17}O(4)$.

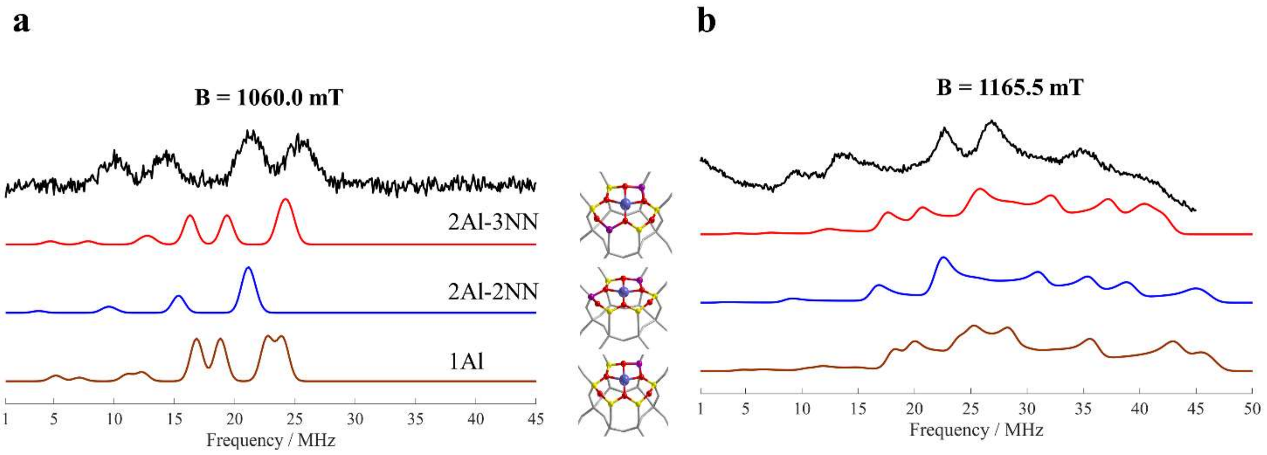

Supplementary Figure 7. Comparison of experimental (black) and simulated Q-band ^{17}O ENDOR spectra of the ^{17}O enriched dehydrated sample. Panel **a** spectra taken at a magnetic field **B**=1060.0 mT; **b** spectra taken at a magnetic field **B**=1165.5 mT. The simulated spectra were obtained by using the computed ^{17}O hyperfine coupling tensors for the three different Al distributions considered in the main text (1Al in brown, 2Al-2NN in blue and 2Al-3NN in red). All four coordinating oxygen donor atoms were considered. We remark that the data refer to geometrically optimized structures at 0 K, while averaged parameters are determined in the experiment.

Models	Nuclei	*d* (Cu-O)	ρ_{spin}	2*s*	$2p_x$	$2p_y$	$2p_z$
$[Cu^{II}(H_2O)_6]$@CHA	O_{e1}	0.210	0.022	0.008	0.010	0.008	0.006
	O_{e2}	0.193	0.056	0.012	0.013	0.009	0.027
	O_{e3}	0.194	0.042	0.010	0.012	0.015	0.010
	O_{e4}	0.209	0.020	0.008	0.016	0.003	0.002
	O_{a1}	0.224	0.004	0.002	0.001	0.002	0.003
	O_{a2}	0.227	0.004	0.001	0.000	0.000	0.004
$[Cu^{II}(H_2O)_4(O-8MR)_2]$	O_{e1}	0.199	0.046	0.011	0.006	0.032	0.006
	O_{e2}	0.206	0.037	0.009	0.001	0.027	0.007
	O_{e3}	0.199	0.044	0.009	0.024	0.006	0.013
	O_{e4}	0.196	0.051	0.011	0.004	0.021	0.022
	O_{a1}	0.234	0.001	0.000	0.000	0.000	0.000
	O_{a2}	0.241	0.001	0.000	0.000	0.000	0.000

Supplementary Table 1. Mulliken spin population analysis and Cu-O bond distances calculated at B3LYP-D3(ABC)/EPR-III(modified) level of theory for $[Cu^{II}(H_2O)_6]@CHA$ and $[Cu^{II}(H_2O)_4(O-8MR)_2]$ periodic models illustrated in Figure 6. The labelling of the oxygen atoms refers to the one indicated in Figure 6. Cu-O bond distances are given in nm.

Models	Nuclei	*d* (Cu-O)	ρ_{spin}	2*s*	2p_x	2p_y	2p_z
2Al-3NN	O_1	0.202	0.084	0.010	0.018	0.062	0.000
	O_2	0.202	0.041	0.008	0.014	0.029	0.000
	O_3	0.195	0.059	0.010	0.055	0.000	0.000
	O_4	0.196	0.044	0.007	0.014	0.025	0.000
2Al-2NN	O_1	0.205	0.104	0.013	0.025	0.076	0.000
	O_2	0.194	0.062	0.009	0.024	0.034	0.000
	O_3	0.195	0.045	0.007	0.041	0.000	0.000
	O_4	0.205	0.030	0.007	0.007	0.022	0.000
1Al	O_1	0.199	0.102	0.012	0.023	0.075	0.000
	O_2	0.203	0.039	0.008	0.014	0.026	0.000
	O_3	0.193	0.067	0.008	0.061	0.000	0.000
	O_4	0.204	0.029	0.006	0.008	0.017	0.000

Supplementary Table 2. Mulliken spin population analysis and Cu-O bond distances calculated at B3LYP-D3(ABC)/EPR-III(modified) level of theory for $[Cu^{II}(O-6MR)_4]$ periodic models at 2Al-3NN, 2Al-2NN and 1Al sites. The labelling of the oxygen atoms refers to the one indicated in Figure 4a. Cu-O bond distances are given in nm.

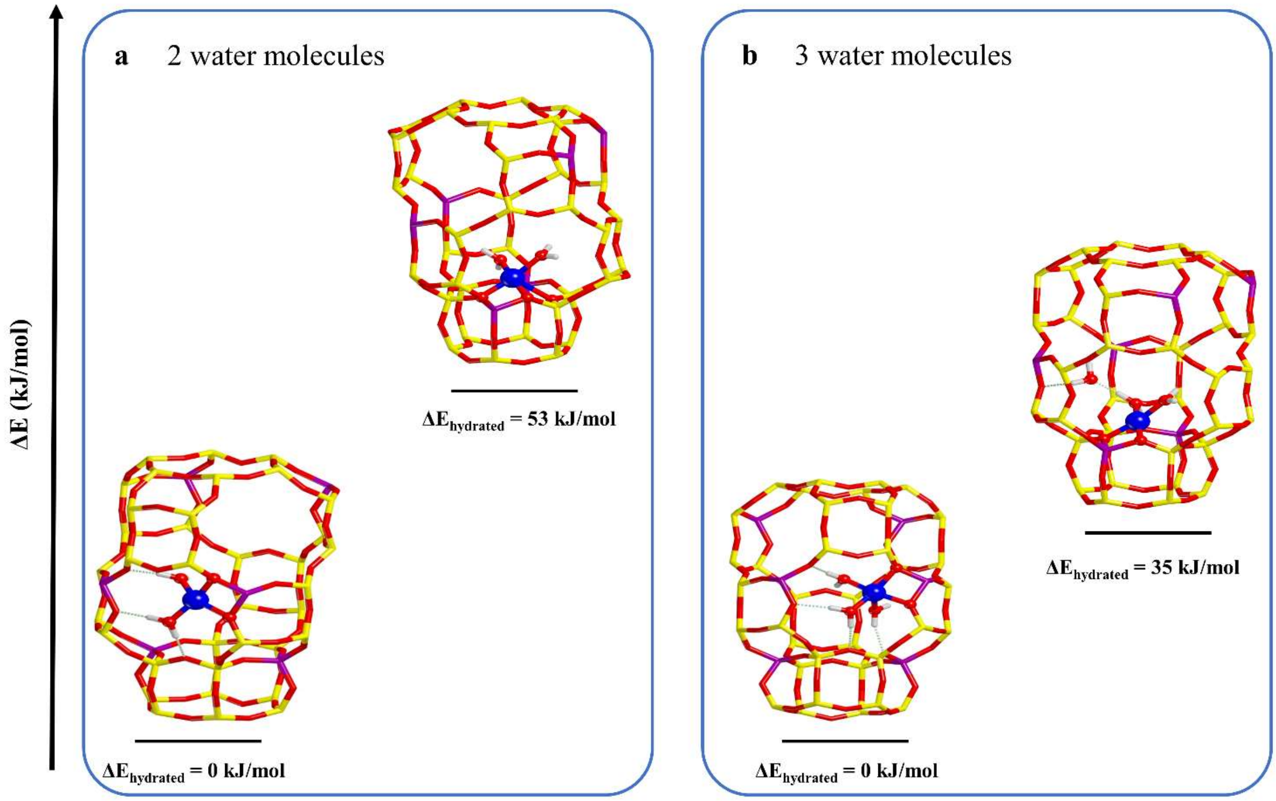

Supplementary Figure 8. Full optimized structures at B3LYP-D3(ABC)/pob-TZVP level of theory of Cu^{II} species sitting in 6MRs and 8MRs hydrated with **a** 2 water molecules and **b** 3 water molecules. Cu are indicated with blue balls, Al with violet balls, O red balls, H in white balls and Si in yellow sticks. The relative stability of both the sites is evaluated in terms of relative electronic energy per unit cell. DFT computations point out that, in presence of more than one water molecules in the copper coordination sphere, 8MR sites appears to be more stable with respect to 6MR location (Supplementary Figure 8). This difference between the two sites is likely due to the lower coordination number of Cu^{II} cations in 8MR in comparison to 6MR sites when no adsorbates are present. Moreover, the adsorption of water molecules provokes significant change in the local geometry of the copper ions (from distorted square planar to a distorted square pyramidal geometries). Note that the third water molecules in 6MR site does not interact directly with Cu but it is linked to its first coordination sphere through hydrogen bonds. The increasing of the water molecules bound to Cu^{II} leads copper ion shifted upward, above the position of 6MR unit. Such findings are in agreement

with the work of Kerkeni (see Reference 71 of the main text) and prove how the location and geometry of copper ions in CHA framework are drastically affected by the presence of adsorbates with oxygen donor atoms like water molecules.

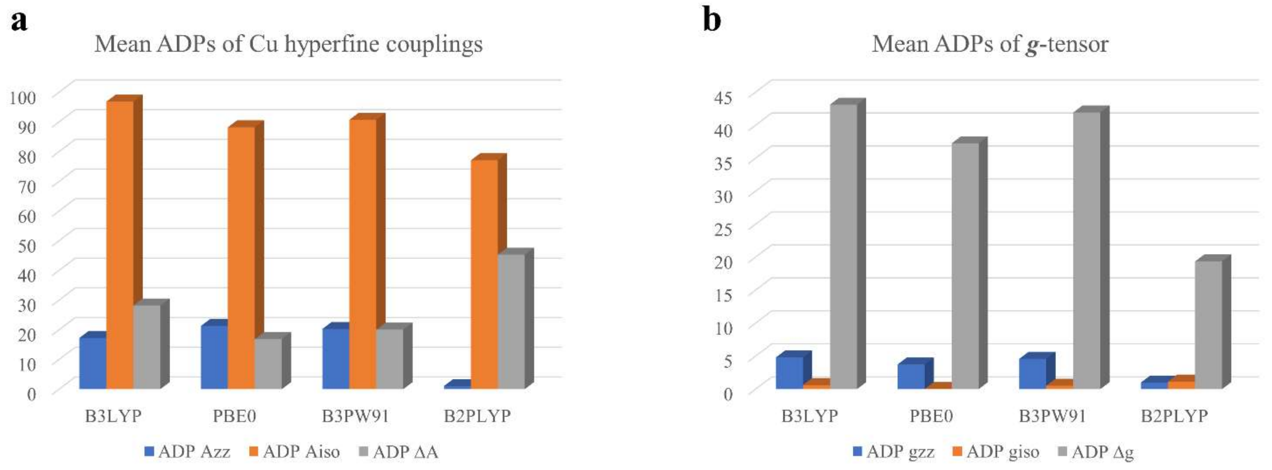

Supplementary Figure 9. Graphical representations of mean ADPs of A_{33}, A_{iso} and ΔA (panel **a**) and g_{33}, g_{iso} and Δg (panel **b**) for calculated values of $[Cu(H_2O)_6]^{2+}$ obtained with CP(PPP) basis set using different functionals with respect the experimental result. We used as reference experimental values the ones found for species A in Table 1, assigned to a $[Cu(H_2O)_6]^{2+}$ complex.

Models		g_{xx}	g_{yy}	g_{zz}	A_{xx}	A_{yy}	A_{zz}
$[Cu^{II}(H_2O)_6]$@CHA	exp	2.070	2.070	2.415	30	30	360
	DFT	2.116	2.131	2.391	188	195	396
$[Cu^{II}(H_2O)_4(O-8MR)_2]$	exp	2.065	2.065	2.370	30	30	450
	DFT	2.072	2.116	2.327	93	189	415

Supplementary Table 3. B2PLYP-D3 computed g and ^{63}Cu hyperfine principal components for hydrated cluster models compared with experimental values taken from Table 1. Reported hyperfine parameters are expressed in MHz. For the sake of clarity, we reported the absolute values of the computed hyperfine components.

Models	g_{xx}	g_{yy}	g_{zz}	A_{xx}	A_{yy}	A_{zz}
2Al-3NN	2.070	2.076	2.282	42	62	455
2Al-2NN	2.060	2.082	2.278	38	83	433
1Al	2.068	2.074	2.275	32	35	469
Experimental	2.058	2.058	2.355	30	30	462

Supplementary Table 4. B2PLYP-D3 computed g and ^{63}Cu hyperfine principal components for $[\mathrm{Cu^{II}(O-6MR)_4}]$ cluster models at 2Al-3NN, 2Al-2NN and 1Al sites compared with signal C taken from Table 1. Signal D was not reported since it represents only the 15 % of the total simulated EPR signal. Reported hyperfine parameters are expressed in MHz. For the sake of clarity, we reported the absolute values of the computed hyperfine components.

Models	*d (Cu-H$_{eq}$)*	*d (Cu-H$_{ax}$)*
$[\mathrm{Cu^{II}(H_2O)_6}]$@CHA	0.27	0.30
	0.28	0.28
	0.25	0.30
	0.25	0.28
	0.26	
	0.26	
	0.29	
	0.26	
$[\mathrm{Cu^{II}(H_2O)_4(O-8MR)_2}]$	0.25	0.30
	0.26	0.29
	0.26	
	0.26	
	0.25	
	0.26	

Supplementary Table 5. Bond distances of Cu-H obtained from $[\mathrm{Cu^{II}(H_2O)_6}]$@CHA and $[\mathrm{Cu^{II}(H_2O)_4(O-8MR)_2}]$ periodic models after fully optimization at B3LYP-D3(ABC)/pob-TZVP level of theory. Equatorial (*eq*) and axial (*ax*) protons are listed distinctly. The distances are given in nm.

Models		^{17}O **A**-tensor					^{1}H **A**-tensor					^{27}Al **A**-tensor					
		a_{iso}	T_1	T_2	T_3	e^2qQ/h		a_{iso}	T_1	T_2	T_3		a_{iso}	T_1	T_2	T_3	e^2qQ/h
(a)	O_{e1}	-40.1	5.1	5.5	-10.6	10.3	H_{eq}	2.0	-2.8	-5.5	8.4						
	O_{e2}	-55.7	11.3	11.2	-22.5	9.0	H_{ax}	0.4	-2.5	-2.7	5.2						
	O_{e3}	-58.7	9.0	8.9	-17.8	10.0											
	O_{e4}	-45.4	4.9	5.1	-10.0	10.3											
	O_{a1}	-8.3	1.8	1.8	-3.7	9.4											
	O_{a2}	-6.9	1.7	1.6	-3.3	9.7											
(b)	O_{e1}	-45.4	9.0	9.0	-18.0	4.9	H_{eq}	1.05	-2.4	-7.7	10.1	Al_1	-1.3	-0.9	-0.5	1.4	9.9
	O_{a2}	0.7	0.8	0.7	-1.5	4.1	H_{ax}	-0.2	-2.3	-2.5	4.8	exp	-2.3	-1.0	-1.0	2.0	≤ 4
	O_{e3}	-46.7	10.3	10.6	-20.9	9.0	exp	-1.5	-4.0	-6.0	10.0						
	O_{e4}	-55.8	9.8	9.8	-19.5	9.3	exp	0.3	-4.0	-3.0	7.0						
	O_{a1}	2.6	0.5	0.6	-1.1	9.3											
	O_{e2}	-42.5	7.9	8.1	-16.0	9.7											
	exp	-44.0	8.0	8.0	-16.0	3.0											
	exp	-10.0	2.0	2.0	-4.0	3.0											

Supplementary Table 6. Periodic computed spin-Hamiltonian parameters of ^{17}O, ^{27}Al and ^{1}H nuclei on (a) $[Cu^{II}(H_2O)_6]@CHA$ and (b) $[Cu^{II}(H_2O)_4(O-8MR)_2]$ atomistic models at B3LYP-D3(ABC) level of theory. The labelling of the atoms refers to the one indicated in Figure 6. Hyperfine coupling constants and quadrupole coupling constants are given in MHz. The experimental values are taken from Table 2 and 3 for comparison. The computed hyperfine values, listed in Supplementary Table 6, evince an exquisite agreement with experimentally obtained tensors. For the sake of clarity, we reported the mean values of a_{iso} and T components of the equatorial and axial protons. While the computed a_{iso} term for axial water protons is always set around 0.1-0.4 MHz, the isotropic hyperfine coupling constants of the equatorial water molecules depend on the orientation of the water molecules with respect to the equatorial plane, as previously discussed by Larsen.[1] Therefore a realistic comparison of the experimental and computed isotropic *hfi* is difficult to assess because the actual orientation of water ligands is affected by remote water molecules as well as the zeolite framework.

Models		^{17}O **A**-tensor						^{27}Al **A**-tensor				
		a_{iso}	T_1	T_2	T_3	e^2qQ/h		a_{iso}	T_1	T_2	T_3	e^2qQ/h
2Al-3NN	O_1	-37.5	16.5	16.4	-32.9	4.1	Al_1	-5.6	0.2	1.3	-1.4	12.3
	O_2	-45.4	8.9	9.0	-18.0	5.2	Al_2	0.5	-0.3	-0.7	1.0	13.1
	O_3	-49.8	11.8	11.8	-23.6	4.6						
	O_4	-36.0	8.6	8.6	-17.2	5.1						
2Al-2NN	O_1	-39.9	20.5	20.3	-40.8	4.1	Al_1	0.9	-0.5	-0.9	1.3	12.7
	O_2	-43.0	12.2	12.1	-24.2	4.6	Al_2	-6.0	0.2	1.2	-1.4	11.7
	O_3	-41.1	9.3	9.4	-18.7	4.5						
	O_4	-37.2	6.7	6.7	-13.4	3.7						
1Al	O_1	-42.1	19.8	19.6	-39.5	4.3	Al_1	-6.6	0.2	1.3	-1.4	16.3
	O_2	-42.8	8.7	8.7	-17.4	5.3						
	O_3	-49.6	12.9	12.9	-25.9	4.8						
	O_4	-32.5	6.3	6.4	-12.7	3.7						
Exp	O(3)	-51.0	12.0	12.0	-24.0	4.0	Al(2)	-3.0	2.0	2.0	-4.0	11.0
	O(4)	-41.0	9.5	9.5	-19.0	4.0						

Supplementary Table 7. Periodic computed spin-Hamiltonian parameters of ^{17}O and ^{27}Al nuclei on $[Cu^{II}(O-6MR)_4]$ periodic models at 2Al-3NN, 2Al-2NN and 1Al sites at B3LYP-D3(ABC) level of theory. The labelling of the atoms refers to the one indicated in Figure 4. Hyperfine coupling constants and quadrupole coupling constants are given in MHz. The experimental values are taken from Table 2 and 3 for comparison.

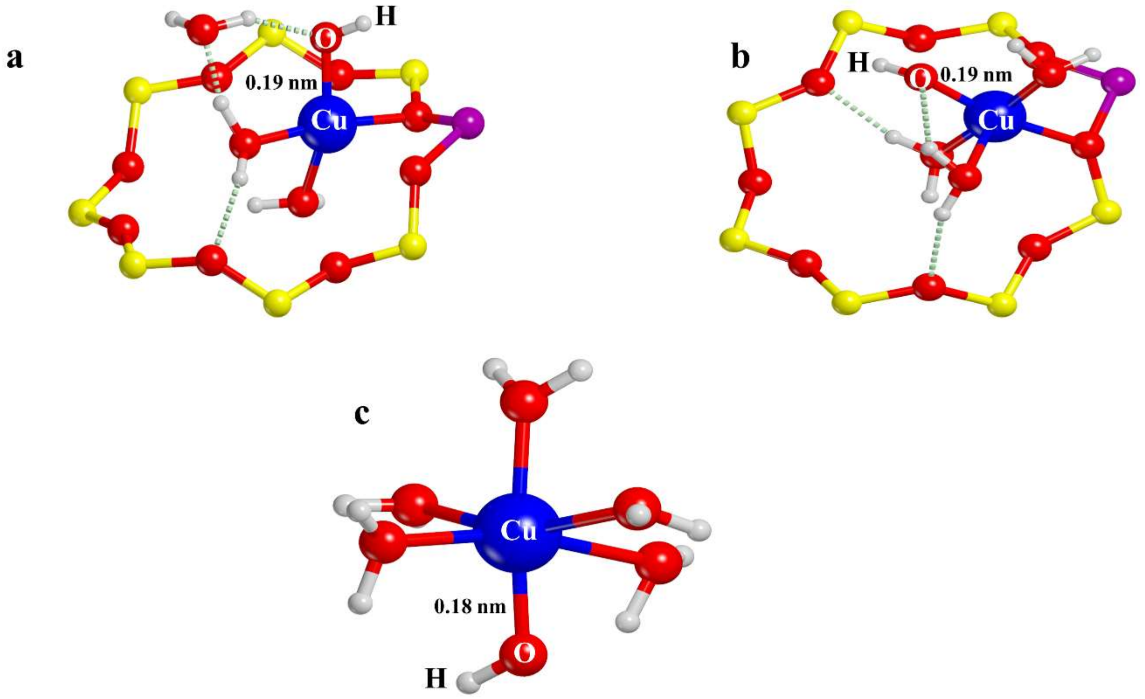

Supplementary Figure 10. B3LYP-D3(ABC)/pob-TZVP fully optimized structure of **a** [$Cu^{II}(OH)(O\text{-}8MR)_1(H_2O)_3$] complex obtained from the substitution of one equatorial water molecule with OH^- in model of Figure 6b, **b** [$Cu^{II}(OH)(O\text{-}8MR)_1(H_2O)_4$] complex obtained from the substitution of one axial water molecule with OH^- in model of Figure 6b **c** [$Cu^{II}(OH)(H_2O)_5$] complex relaxed inside the biggest cavity of CHA framework. Cu-O bond length involving the OH^- group are reported in bold. Al atoms are in violet, H white, Si in yellow, O in red and Cu in blue.

Model	Nucleus	a_{iso}	T_1	T_2	T_3	e^2qQ/h
(a)	^{1}H	-8.6	-3.1	-15.6	18.6	
	^{17}O	-34.8	26.0	25.7	-51.7	7.9
(b)	^{1}H	-2.7	-3.0	-15.8	18.8	
	^{17}O	-40.2	25.6	25.9	-51.5	8.0
(c)	^{1}H	-7.7	-2.4	-16.5	18.9	
	^{17}O	-31.1	27.0	26.7	-53.7	7.3

Supplementary Table 8. Periodic computed spin-Hamiltonian parameters of ^{17}O and ^{1}H nuclei related to the OH^- group of atomistic models shown in Figure S10 at B3LYP-D3(ABC) level of theory. Hyperfine coupling constants and quadrupole coupling constants are given in MHz.

Supplementary Note 1

From the observation of the hyperfine structure and determination of the isotropic (a_{iso}) and dipolar (T) hyperfine couplings, the electronic spin distribution in a molecular fragment can be obtained. To do so it is important that the *hfi* interaction with several (preferably all!) nuclei in the molecule are observed. Then, with the knowledge of a_{iso} and T for the atomic species, and assuming that the *hfi* interaction at a given nucleus is proportional to the electron spin density at that nucleus, one can obtain the spin population in s-type orbitals ρ_s, p-type orbitals ρ_p.

For an unpaired electron (free electron, g_e=2.0023) on a ^{17}O nucleus with a unitary spin population (ρ_s =1) in an s-type orbital, one would observe an isotropic hyperfine coupling constant of a_0=-4622.83 MHz. If the electron resides in a *p*-type orbital one would observe a uniaxial hyperfine constant of b_0=130.4 MHz. Including a correction for the difference in the *g* values, the spin populations in *s*-type and *p*-type orbitals can thus be estimated as:

$$\rho_S = \frac{A_{iso}}{a_0}\frac{g_e}{g_{iso}};\ \rho_p = \frac{T}{b_0}\frac{g_e}{g_{iso}}$$

In the calculation of ρ_p we consider that the value used for T should be corrected for the through space dipolar interaction (T^d) between the magnetic moment of ^{17}O and spin population that is located on the metal center. Using the Cu-O values obtained from the DFT calculations and assuming 70% spin density on Cu, for the hydrated case, we can estimate the contribution to the ^{17}O-hyperfine matrix as T^d = [0.94,0.94, -1.88] MHz for the equatorial O ligand ($d_{Cu\text{-}O}$ =0.20 nm) and T^d = [0.54, 0.54, -1.08] MHz for the axial O ligands ($d_{Cu\text{-}O}$ =0.24 nm) respectively. The corrected value to estimate ρ_p is thus given by: T=T_{tot}- T^d= 8.00 -0.94 MHz=7.06. MHz for the equatorial case and T=T_{tot}- T^d= 2- 0.54 MHz=1.46 MHz for the axial case, corresponding to 5.4% and 1.1% O *p* character for the equatorial and axial ligands, respectively. Analogous calculations in the case of the square planar complex of the dehydrated system lead to ρ_p=8.5% and ρ_p=6.6% for the two oxygen families ($^{17}O(3)$ and $^{17}O(4)$ in Table 3 of the main text). The total spin density on the oxygen ligands obtained as the sum of the two ρ_s and ρ_p contributions is reported in the main text. These values nicely agree with the Mulliken spin population reported in Supplementary Tables 1 and 2.

Supplementary Note 2

The precise and robust calculation of **g**- and **A**-tensors for mononuclear Cu^{II} systems is still a great challenge for quantum chemistry methods.[2,3] While for organic radicals excellent results for **g**-tensor and hyperfine couplings are already obtained with GGA functionals, for transition metal ions the situation is totally different. On one hand, *g*-shifts are usually underestimated by standard DFT methods because of a too covalent description of the metal-ligands bonds and a overestimation of the *d-d* transition energies.[4] On the other hand, the problems in the prediction of hyperfine couplings are related to the spin-orbit coupling (SOC) component (negligible for lighter elements) and to the Fermi

contact which depends on the indirect core level spin polarization arising from the unpaired spin density in the metal *d*-orbitals. Again, these two terms are somehow underestimated by common DFT methods.

Although hybrid functionals (like PBE0, B3LYP, B3PW91) are usually to be preferred in terms of average errors,[4] there is no first-rate choice able to provide steady and general performance. Besides, the advent of wave function-based methods, accessible thanks to the development of algorithmic approximations, has recently proven to grant more accurate and systematic results.[5,6]

Fully aware of this, we tested the performance of B3LYP, PBE0, B3PW91 hybrid functionals and B2PLYP double-hybrid method in conjunction with CP(PPP) basis set for Cu in computing **g**- and **A**-tensors of Cu^{II} ion in our easiest model, the $[Cu(H_2O)_6]^{2+}$ complex. To evaluate the performance of the methods we assessed absolute percent deviations (APD) for g_{zz}/A_{zz} (largest components of **g**- and **A**-tensors, Equation 1), g_{iso}/A_{iso} (defined as the average of $[g_{xx}/A_{xx} + g_{yy}/A_{yy} + g_{zz}/A_{zz}]/3$, Equation 2) and $\Delta g/\Delta A$ (defined as the difference g_{max}/A_{max} - g_{min}/A_{min}, Equation 3). Then the ADPs were evaluated as follows:

$$ADP\ (A,g)_{zz} = \left| \frac{(A,g)_{zz}^{calc} - (A,g)_{zz}^{exp}}{(A,g)_{zz}^{exp}} \right| \times 100 \tag{1}$$

$$ADP\ (A,g)_{iso} = \left| \frac{(A,g)_{iso}^{calc} - (A,g)_{iso}^{exp}}{(A,g)_{iso}^{exp}} \right| \times 100 \tag{2}$$

$$ADP\ \Delta(A,g) = \left| \frac{\Delta(A,g)^{calc} - \Delta(A,g)^{exp}}{\Delta(A,g)^{exp}} \right| \times 100 \tag{3}$$

Regarding the prediction of hyperfine couplings, our results indicate an enormous deviation from the experimental value of A_{iso}, whereas **g**-tensor are generally better predicted, apart from the Δg parameter (Supplementary Figure 9). This is due to an overestimation of g_{xx} and g_{yy} with respect to the experimental values.

Overall, B2PLYP method performs better with respect to the other hybrid functionals, in particular for computation of **g**-tensor. Among the hybrid functionals tested, B3PW91 appears to give better results for hyperfine couplings calculation (in agreement with the work of Gómez-Piñeiro *et al.*)[7] whereas PBE0 works better for the prediction of **g**-tensor.

Supplementary Note 3

The hyperfine couplings of ^{1}H and ^{17}O from OH^- group linked to Cu^{II} aqua complexes attached or completely detached from the CHA framework were computed (see Supplementary Figure 10). Model (a) was built by starting from the optimized structure of $[Cu^{II}\ (H_2O)_4(O\text{-}8MR)_2]$ (Figure 6b) and substituting one equatorial water with a OH^- group and one Al atom with Si. Analogously, model (b) was obtained by replacing one axial water with a hydroxyl group. Finally, model (c) is $[Cu^{II}(OH)(H_2O)_5]$ complex optimized inside the biggest cavity of CHA framework. We assumed to

use as guessing point Cu complex with octahedral geometry since **g**-tensor and Cu hyperfine couplings measured for the hydrated state are consistent with such coordination. However, the full optimized structures of (a) and (b) assume a distorted tetragonal and square pyramidal geometry, respectively. On the contrary, model (c) keeps a distorted octahedral geometry. In any case, the Cu-O bond related to the hydroxyl group is slightly shorter with respect to the Cu-O bonds involving water molecules. This is fundamentally due to the stronger negative charge of the oxygen atom in OH^- group with respect to water ligands. The computed hyperfine ^{1}H and ^{17}O couplings of the hydroxyl group are completely out of the range of the experimental values (see Supplementary Table 6 and Tables 2 and 3), especially the dipolar part. These findings confirm the proposed assignment for the experimental ^{1}H and ^{17}O signals in HYSCORE and ENDOR spectra. Moreover, we also prove that the combination of hyperfine techniques with computational modelling can be exploited to distinguish hydrated $[CuOH]^+$ species from aqua Cu^{II} complexes in hydrated copper-exchanged zeolites.

Supplementary References

1. Larsen, S. C. DFT calculations of proton hyperfine coupling constants for $[VO(H_2O)_5]^{2+}$: Comparison with proton ENDOR data. *J. Phys. Chem. A* **105**, 8333–8338 (2001).

2. Neese, F. Metal and ligand hyperfine couplings in transition metal complexes: The effect of spin-orbit coupling as studied by coupled perturbed Kohn-Sham theory. *J. Chem. Phys.* **118**, 3939–3948 (2003).

3. Neese, F. Prediction of molecular properties and molecular spectroscopy with density functional theory: From fundamental theory to exchange-coupling. *Coord. Chem. Rev.* **253**, 526–563 (2009).

4. Kaupp, M. *et al.* Calculation of electronic g-Tensors for transition metal complexes using hybrid density functionals and atomic meanfield spin-orbit operators. *J. Comput. Chem.* **23**, 794–803 (2002).

5. Tran, V. A. & Neese, F. Double-hybrid density functional theory for g-tensor calculations using gauge including atomic orbitals. *J. Chem. Phys.* **153**, 54105 (2020).

6. Shiozaki, T. & Yanai, T. Hyperfine Coupling Constants from Internally Contracted Multireference Perturbation Theory. *J. Chem. Theory Comput.* **12**, 4347–4351 (2016).

7. Gómez-Piñeiro, R. J., Pantazis, D. A. & Orio, M. Comparison of Density Functional and Correlated Wave Function Methods for the Prediction of Cu(II) Hyperfine Coupling Constants. *ChemPhysChem* **21**, 2667–2679 (2020).
